# Lived Experiences of Migrant Fathers in the Perinatal Period: A Systematic Review and Analysis

**DOI:** 10.1007/s10903-024-01627-0

**Published:** 2024-08-29

**Authors:** Huy N. Vo, Kirstie McKenzie-McHarg, Pauleen C. Bennett, Dac L. Mai

**Affiliations:** Department of Psychology and Counselling, School of Psychology and Public Health, La Trobe University, Flora Hill, Bendigo, VIC 3552 Australia

**Keywords:** Perinatal care, Migrant fathers, Lived experiences, Mixed-methods

## Abstract

The worldwide population of migrant families is on the rise, and there is growing acknowledgement of the significance of supporting parental mental health within these families. However, understanding of the experiences of migrant fathers during the perinatal period remains incomplete. The objective of this review is to provide an overview of existing research on perinatal migrant fathers’ experiences in different cultures. Multiple searches were conducted in April 2023 for quantitative, qualitative, and mixed-methods studies across six electronic databases: Medline, CINAHL, Embase, PsycINFO, Web of Science, and Scopus. Fourteen eligible articles were identified, including nine qualitative studies, five quantitative studies, and no mixed-methods studies. The Mixed-methods Appraisal Tool was used to assess the quality of these studies. The quantitative findings were transformed into narrative summaries to be analysed thematically along with the qualitative data. Three themes were identified: (1) Cultural competence (dealing with cultural differences, needs related to original country); (2) Parenthood in a new country (challenges and adaptation to fatherhood, challenging traditional gender norms, lack of extended family and building new support networks, being the main supporter for the family); (3) Needs of the fathers and their personal difficulties. The findings of this review suggest a direction for future research in perinatal psychology. The review also provides insights into the need for social and community support for migrant fathers and how healthcare services can support this group during the perinatal period.

**Supplementary Information:**

The online version contains supplementary material available at 10.1007/s10903-024-01627-0.

Fathers play many important roles in the family life cycle, including providing support during their partner’s pregnancy and during the first year postpartum, referred to as the perinatal stage [1]. Although families exist in many configurations, for the purposes of this review we defined fathers as men with a perinatal partner, regardless of the nature of their cohabitation. Given the vital role of fathers during the perinatal period, it is reasonable to suggest that this responsibility may place additional pressures on them, potentially impacting their mental well-being and their ability to offer sustained support to their families [2]. These effects may be further pronounced among fathers who are first-generation immigrants or refugees in a new country, whom we will simply refer to as migrant fathers in this review. Within the new family dynamic, those fathers often juggle being the primary breadwinner and caregiver while also dealing with the stresses of adapting to a new culture [3]. Although there have been numerous systematic reviews and even umbrella reviews of research focusing on mothers [4–6], reviews of perinatal research focusing on fathers have emerged only recently [7]. A systematic review by Mprah et al. looked into the experiences of fathers in general, including research mostly on non-immigrant fathers, with the experiences of immigrant fathers being addressed only minimally [8]. To extend on the work of Mprah et al., the current paper concentrates particularly on the lived experiences and cultural challenges faced by migrant fathers living in a new country.

During the perinatal period, fathers encounter a range of mental health challenges. Numerous studies have yielded valuable insights into the mental and psychological well-being of fathers during pregnancy, childbirth, and transition to fatherhood. Wong, Nguyen [9], found that the risk of psychological distress among fathers during the perinatal period was notably high. Anxiety was common, with up to 25% of fathers experiencing anxiety during the antenatal period and up to 51% during the postnatal period [10]. Furthermore, the prevalence of fathers experiencing depression during the entire perinatal period was estimated at 8.4% [11].

Most research has predominantly focused on data from native fathers, leaving a gap in psychological research concerning the mental health of migrant fathers. In 2023, the systematic review by Mprah et al. [8] captured the experiences of perinatal fathers, including migrant fathers, regarding their interactions with healthcare systems and their involvement in maternity experiences. However, the search terms employed were broad and the inclusion and exclusion criteria were not reported in much detail. The author included only two studies that cover migrant fathers in their general review, suggesting that a more focused review might also be of value. With a focus particularly on the experiences of migrant new fathers, our preliminary library searches identified a few papers that were not included in that review, justifying this additional contribution.

According to Bond [12], most research on migrant fathers has been conducted in developed countries, focusing on fathers from developing countries [12]. This could be because those who migrated from countries with similar cultural or socioeconomic backgrounds were found to be less stressed than those who came from different backgrounds of the host country [13, 14]. Although the reasons for migration are diverse, but the two most prevalent are unfavourable economic and political conditions in their country of origin [15]. In a new country, immigrants seek access to higher-quality education, healthcare, and employment opportunities. Successful migration requires significant effort to adjust to a new setting compared to their country of origin [16]. Migrant fathers in the perinatal period, in particular, encounter numerous challenges during the transition to fatherhood as they navigate the demands of acculturation, adapting to cultural norms and values different from their homeland. Several studies in various countries highlight the lived experiences of these fathers, emphasizing the pressure they face to become the primary support for their families in a new context, balancing traditional demands with host country expectations [12, 17–19].

Having to deal with multiple stressors at the same time is a known risk factor for developing psychological issues for migrant fathers [12]. This may be amplified further when these multiple stressors meet at an intersection. Migrant fathers often have to carry out roles that are different from the expectations of their heritage culture. For instance, many migrant fathers came from countries where gender norms are strictly defined based on biological sex [17]. These norms can impose restrictive expectations on how fathers are perceived and portrayed, for instance, discouraging their involvement in childcare, and considering it as a woman’s role [19]. However, when settling in a new country, these fathers may adjust by trying to share equal responsibilities with their wives [20].

In short, migrant fathers may be at a higher risk of experiencing psychological distress as they experience stressors from both their transition to a new country and their new fatherhood role [21]. Researchers have started to look at the lifeworld of migrant fathers, particularly the well-being aspect during the perinatal period of their partner. Although those aspects show a spectrum of lived experiences of these fathers, no systematic review has synthesised these multifaceted experiences of this specific population in this critical period. Therefore, the objective of this review is to systematically identify, evaluate, and synthesize published literature on the lived experiences of first-generation migrant fathers, defined as individuals born in one country who have moved to another country to live, work, or study.

## Method

This study was pre-registered on the Open Science Framework [22] and, to improve transparency, we followed the PRISMA statement [23] and eight QESISAES stages [24]. In addition, the SPIDER framework [25] was used to develop review questions and strategies for searching.

### Sources and Search Strategy

A systematic search was conducted in six databases: Medline, CINAHL, Embase, Scopus, PsycINFO, and Web of Science. The original search terms were developed by the research team based on the SPIDER statements specific to this review (refer to Table 1), which were then reviewed by an experienced librarian from La Trobe University. The final search terms and Boolean operators are listed in Table 2. The search was performed in April 2023, and no time restrictions were imposed on publication dates.

**Table 1:** SPIDER statements in the present review

SPIDER	Element	Focus of present review
S	Sample	Migrant fathers
P, I	Phenomenon of interest	Perinatal period
D	Design	There are no restrictions imposed on the research design
E	Evaluation	Subjective and objective data regarding the lived experiences of migrant fathers
R	Research type	Quantitative, qualitative, or mixed-methods studies

**Table 2:** Visual depiction of search terms and boolean operators used in the search strategy

Search Terms
“Father*” OR “men” OR “dad*” OR “paternal” OR “male” OR “male parent” OR “fatherhood”
**AND**
“immigration” OR “migration” OR “migrant” OR “immigrant” OR “refugee” OR “asylum” OR “accultu*”
**AND**
“postnatal” OR “postpartum” OR “puerperium” OR “perinatal” OR “postpartum care” OR “postnatal care” OR “perinatal care” OR “pregnancy care” OR “breastfeeding” OR “newborn” OR “infant” OR “baby” OR “birth” OR “partner support” OR “parent*” OR “prenatal” OR “antenatal”
**AND**
“mental health” OR “stress” OR “depress*” OR “anxiety” OR “happ*” OR “growth” OR “trauma” OR “experience*”

### Criteria for Inclusion and Exclusion

The eligibility criteria included being written in English, peer-reviewed, and focused on perinatal care (from early pregnancy to 12 months postpartum) with the inclusion of migrant fathers. If the studies also included participants other than new fathers (e.g., mothers and midwives), the results should clearly show the lived experiences of the fathers. The review exclusively extracted data about fathers by examining each paper line by line. Whenever the authors identified a sentence about fathers, that sentence was included in the pool for later analysis. We only chose studies that analysed primary data and were presented in a full-text format. Opinion pieces, guidelines, case studies, conference presentations, reviews, and unpublished research papers were excluded.

### Quality Appraisal and Risk of Bias Assessment

The first author (Vo) imported the search results into Covidence (covidence.org), an online platform that facilitates systematic reviews. Within this platform, duplicate studies were removed, and the titles and abstracts of the remaining studies were screened based on predetermined inclusion and exclusion criteria. Throughout this process, author Vo maintained ongoing discussions with author Mai regarding the selection of studies. Subsequently, a total of forty-three papers were initially considered, and their eligibility was deliberated among all four authors.

Author Vo evaluated the quality of the 14 selected studies using the Mixed Methods Appraisal Tool (MMAT; Hong et al., [26]). This tool is recommended for the appraisal of quantitative, qualitative, and mixed-methods research designs, focusing on their methodological quality criteria, such as sampling strategies, data analysis, and measurement appropriate. All selected studies were qualified to be analysed in the next step and the appraisal of each study is shown in Table S1 in the Supplementary Materials.

Following the identification of the 14 selected studies, we utilized a template created by the Toolkit for Mixed Studies Reviews [24]. This template outlines eight key areas that need to be reported in a mixed-methods review: the study author(s), year of publication, country, study design, aim, participants, method of data collection, and findings. In addition, the author also included the strengths and limitations of each study to provide a comprehensive appraisal of the selected studies (Table 3). After reporting and appraising the studies, they were entered into NVivo (version 20) for data extraction, synthesis, and analysis.

**Table 3:** Characteristics and critical evaluations of the reviewed studies

S/N	Author (Year), Country	Aim	Participants	Data analyses	Findings	Strengths	Limitations
*Qualitative studies (n = 9)*							
1	Lee [32], The United States	- To explore the perinatal beliefs and practices of Korean immigrant couples in the U.S. sociocultural context - To investigate the upsides and challenges of Korean immigrant couples’ childbirth experiences in the U.S - To identify sociocultural perinatal health issues of Korean immigrant couples	Korean Couples in the US	Thematic analysis	- Various factors interact to shape how Korean immigrant couples practice the Taekyo and Sanhujori traditions - Korean immigrant couples found positive aspects in their experience, such as personal growth, shared meaningful life events, local community connections, and healthcare benefits	- Analysed data from couples as a unit of research - Collected data using the Korean language	- Participants were only located in the state of Washington - 87% of participants were Christians with at least a bachelor’s degree - All participants had extended family members to support their families
2	Nges et al. [36], Sweden	- To examine the experiences of Cameroonian fathers during their women’s labour and childbirth in Sweden	Cameroonian fathers living in Sweden	Thematic content analysis	- Ambivalent feelings to accompany their women into the maternity unit, an unfamiliar area - Cooperation and finding some places in a foreign area - Knowledge, insight, and transition to fatherhood	- Was the first study to explore the experience of attending childbirth of Cameroonian fathers in Sweden	- Conducted only in 3 cities of Sweden - Did not control for ages, modes of birth, or the number of times fathers experienced childbirth - Only focused on the childbirth experience in a hospital
3	Ny et al. [30], Sweden	- To capture the experiences of Middle Eastern men as they embrace fatherhood in Sweden	Middle East men living in Sweden	Thematic analysis	- Meeting caring and understanding experts - Navigating changing family roles and adapting to evolving social expectations	- Investigated three aspects: maternal and child healthcare and fatherhood - Conducted in the participants’ mother tongue	- Interviewing in groups may have resulted in some individuals feeling dominated
4	Valdez & Martinez [40], the United States	- To investigate how fathers in a Mexican immigrant sample recognize, interpret, and cope with their partners’ maternal depression	Mexican immigrant fathers in the United States	Thematic analysis	- Fathers’ initial understanding of maternal depression - Accurate information about depression enabled fathers to play a more active and supportive role in their families	- Conducted in the Latino language - Used dual-method qualitative research	- Most of the participants work in labouring jobs - Did not report the level of depression of wives - Only a small number of follow-up interviews were conducted
5	Wojnar [31], the United States	- To investigate Somali couples’ viewpoints on the care and support provided during the perinatal phase in the United States	Somali Couples in the United States	Descriptive phenomenological analysis	- Experiencing vulnerability, lack of information, and misunderstanding - Yearning for unconditional respect and acceptance - Navigating survival and growth	- Used semi-structured interviews with couples - Conducted follow-up interviews	- The socioeconomic status and ability to use English of participants varied - Focused more on perinatal experiences in the hospital and healthcare system, not as much on the well-being of the father in the perinatal period
6	Hunter-Adams [35], South Africa	- To examine the role of supporting migrants in their perinatal period when they live in Cape Town, South Africa	Immigrant males and females from Somali, Zimbabwean, and Congolese living in South Africa	Thematical analysis	- The absence of extended family members from Somali, Zimbabwean, and Congolese - Limited connection to the extended family via phone and text messaging - Perceptions of extensive birth support in countries of origin - Lack of social support in South Africa but have new independence	- Had many participants of both genders	- Group interviews may have caused participants to feel dominated - Interviews were conducted in a group format, not with couples
7	Onyeze-Joe et al. [33], Belgium	- To investigate how exposure to Belgian fatherhood norms might reshape the parenting practices of first-time African fathers living in Belgium	African First-time Fathers in Belgium	Thematic narrative analysis	- Keeping the balance of their traditional provider role with breaking gender norms in African culture - Assuming the primary role of offering prenatal and postnatal support while sharing childcare responsibilities with their partner	- Focused solely on the experiences of first-time fathers	- Some interviews may have been affected by the presence of their partner - Conducted during the COVID-19 pandemic
8	Forbes et al. [37], Australia	- To understand the experiences, attitudes, and beliefs about Ethiopian father’s inclusion in perinatal healthcare	Male and female all born in Ethiopia and currently living in Australia	Thematic analysis	- Comparison between Ethiopia and Australia and the social context around birth - Mixed experiences of perinatal healthcare - Diverse experiences with cultural competency among healthcare staff - Perceptions regarding male partner involvement during the perinatal period and healthcare	- Collected data from couples	- Used a snowball method, resulting in participants from the same community - Focused only on the topic of healthcare system
9	Riggs et al. [34], Australia	- Investigates the experiences of Afghan men with their perinatal partner living in Australia	Afghan women and men living in Australia	Thematic analysis	- Playing a crucial role in supporting their wives during pregnancy and postnatal care - Needs of perinatal fathers in the healthcare setting	- Collected data from the fathers’ partners and health professionals	- Lacked consideration for cultural aspects and psychological issues during data analysis
*Quantitative studies (n = 5)*							
1	Capps et al. [38], The United States	- To study how acculturation influences Chinese and Mexican immigrant fathers’ engagement with their infants	Chinese- and Mexican-Origin fathers in the United States	Linear regression	- Chinese fathers with U.S. citizenship show a negative correlation with warmth activities with their infants - English language usage among Mexican fathers is positively linked to physical care and nurturing activities	- Had a large number of participants - Measured acculturation in various aspects using different scales	- Data is from the first wave of a longitudinal study - The scales need to be validated
2	Roubinov et al. [41], the United States	- To measure early father involvement—paternal engagement, accessibility, and responsibility—tailored to encompass essential cultural values pertinent to the paternal role within Mexican-origin families	Mexican-origin fathers living in the United States	Factor analyses	- Mexican-origin fathers engage significantly in direct interaction with their infants - Behavioral responsibility showed a significant correlation with engagement and positive machismo - Aspects of the romantic relationship, cultural orientation, and maternal employment status were connected to measures of father involvement	- Used culturally adapted assessments - Collected data from couples	- The study is a sub-study involving longitudinal research
3	Khalil et al. [39], the United States	- To examine the associations among acculturative stress, perceived stress, social support, and postpartum depression (PPD) symptoms in immigrant Arab American couples	American Arab couples	Bivariate regression	- Paternal and maternal acculturative stress was moderately associated with maternal PPD symptoms (*r* = .39, and 0.46, respectively; p <. 05) - Maternal and paternal acculturative stress demonstrated a correlation (*r* = .61, *p* < .001)	- Collected data from couples	- Had a small sample size - Did not control SES, childhood maltreatment of participants - Focused more on mothers
4	Khalil et al. [43], the United States	- To investigate the link between psychosocial stress variables in mothers and fathers - To explore the correlation between psychosocial aspects in both parents (acculturative stress, anxiety, and depression) and infants’ hair cortisol concentration within immigrant Arab American families	Arab American triads (mother-father-infant)	Pearson correlation and independence group t-tests	- Anxiety symptoms were reported significantly by parents (33% of mothers; 45% of fathers) as well as depression (33% of mothers; 35.5% of fathers) - Paternal acculturative stress, anxiety, and depressive symptoms showed a significant correlation with infants’ hair cortisol concentration - Acculturative stress, anxiety, and depressive symptoms were notably correlated within mother-father dyads	- Used triad data (mother-father-infant) - Investigated stress levels with biological markers	- Was a pilot study with a limited sample size - Some participants had experienced severe trauma - The study’s analysis and discussion focused on mothers - Infants’ ages range from 4 to 24 months old, so some participants were beyond the perinatal period
5	Khalil et al. [42], the United States	- To compare telomere length in mothers, fathers, and infants - To explore how maternal and paternal psychosocial factors relate to their telomere length - To investigate the link between maternal and paternal psychosocial factors and infants’ telomere length among Arab American immigrants	Arab American triads (mother- Father-infant)	Paired-t-tests and linear regression	- Maternal telomere length (TL) had a positive correlation with infants’ TL (*r* = .31, *p* = .04) and was notably shorter (*p* < .001) - Paternal TL did not correlate with infant TL but was significantly shorter than the infant’s TL (*p* < .001) - Maternal depression showed a significant correlation with mothers’ TL (*r* = .4, *p* = .007)	- Provided information about mental health in a biological sense with triadic data collection	- There was no control for body mass index (BMI) or smoking history

### Data Synthesis

Because each study had distinct research questions, study designs, and findings, it was not feasible to conduct a quantitative data analysis or directly compare these studies. Instead, a narrative synthesis approach was employed to summarize the diverse findings in a structured manner [27]. The study followed a convergent synthesis design for the mixed studies review, based on the methodology proposed by Pluye and Hong [28]. This allowed for the conversion of the results from qualitative, quantitative, and mixed-methods studies into qualitative findings. Within NVivo, the primary author extracted all results related to the lived experiences of migrant fathers. Throughout the synthesis and analysis phases, the primary author engaged in discussions with the other three authors to finalize the synthesis and analysis of the selected papers.

Thematic analysis, following the framework by Thomas and Harden [29], was used to synthesize the data. According to the two authors, the meta-synthesis process consisted of three stages: line-by-line coding, developing descriptive themes, and generating analytical themes. Firstly, codes derived from selected articles were extracted based on the research question about the lived experiences of the migrant fathers living with their perinatal partner. Subsequently, the codes were organized into similar areas, resulting in the formation of descriptive themes. Finally, in the third stage, a tree structure with various layers was created, based on the descriptive themes. The participant quotes and the author’s interpretations of each theme are included in Table S2 in the Supplementary Materials.

## Results

### Search Outcomes

**Fig. 1:**
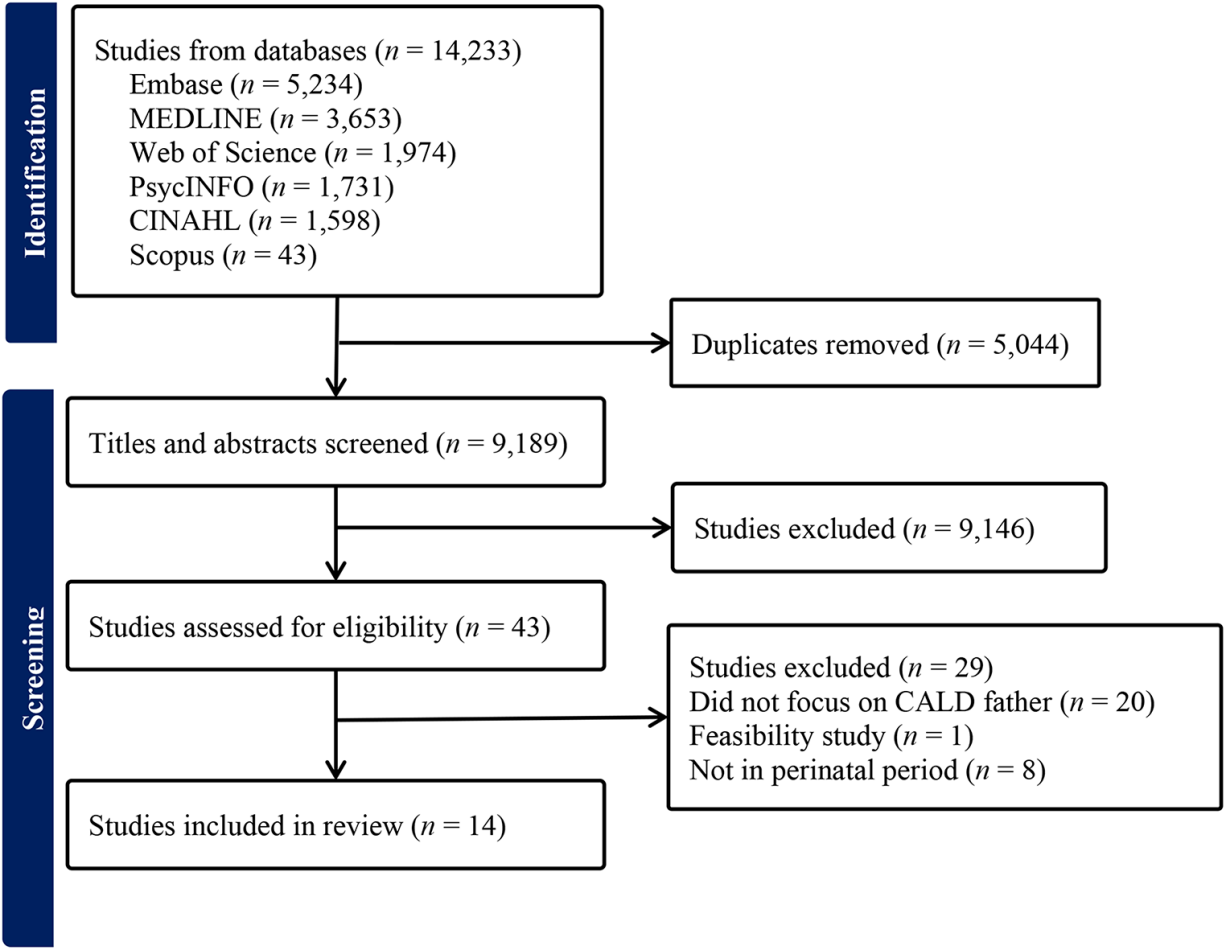
PRISMA flow diagram

A total of 14,233 studies were retrieved from the six databases. After removing 5,044 duplicate research articles, 9,189 studies were screened based on the suitability of titles and abstracts according to the study criteria. Subsequently, 43 studies underwent a full-text review to assess their eligibility. Following the review and discussion by two researchers (Vo and Mai), 14 articles were selected for this review. Figure 1 presents the PRISMA flow diagram illustrating the search and selection outcomes.

### Study Characteristics

Of the 14 included studies, nine were qualitative and five were quantitative studies. The majority of the studies were conducted in the United States (*n* = 8), followed by Europe (Sweden, *n* = 2; Belgium, *n* = 1), Australia (*n* = 2), and South Africa (*n* = 1). In total, this systematic review encompassed the experiences of 1,183 fathers, living with their perinatal partners, from 16 origins.

### Overview of the Thematic Results

**Fig. 2:**
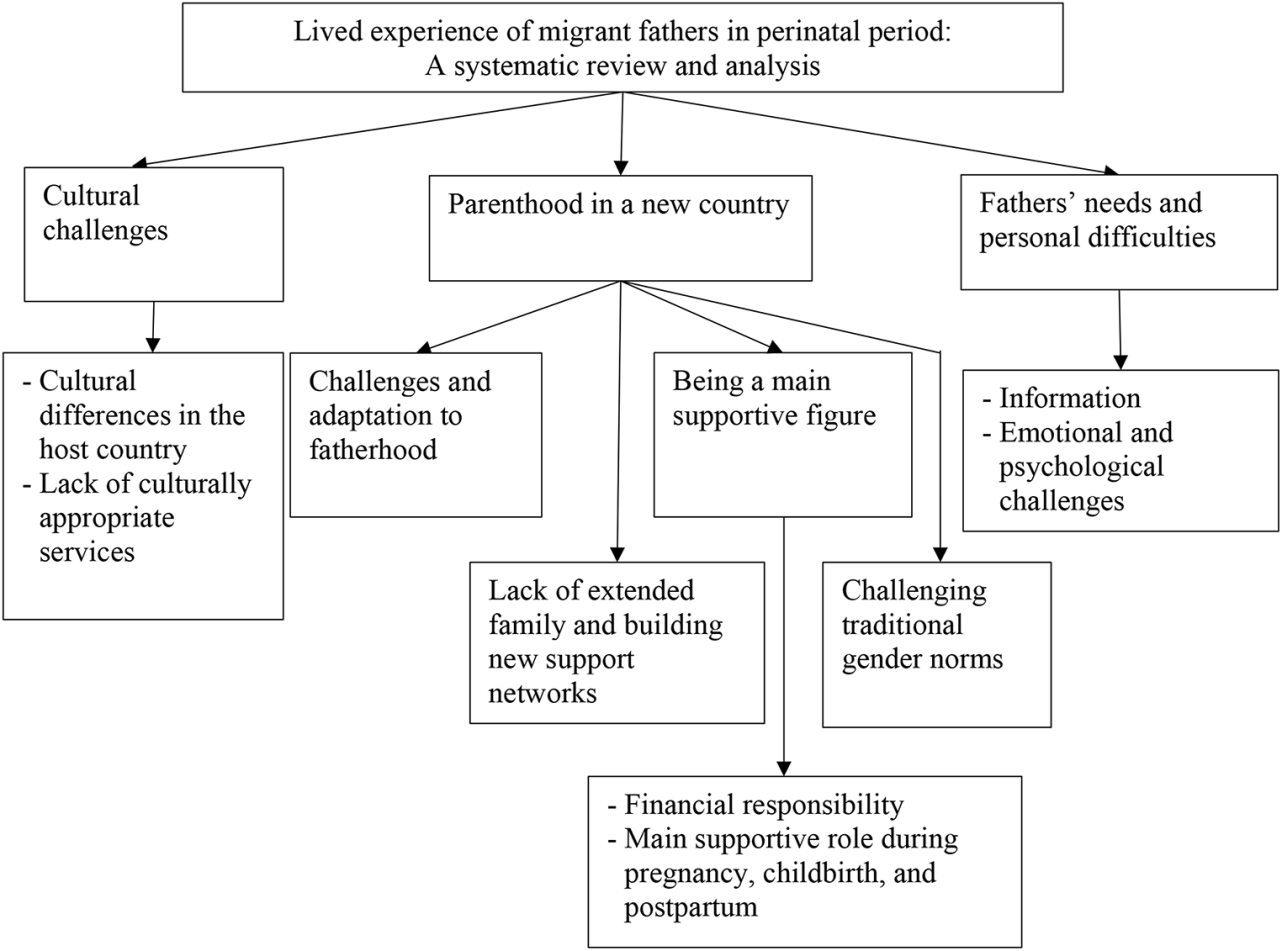
Themes and sub-themes of the present study

Three themes were identified: (1) Cultural challenges, (2) Parenthood in a new country, (3) Fathers’ needs and personal difficulties. Figure 2 presents an overview of the themes and subthemes identified, which are described with illustrative quotes in the following sections.

### Cultural Challenges

As shown in Table 4, eight qualitative and three quantitative studies provided data on new fathers’ experience of cultural challenges. In the synthesis, two subthemes emerged: cultural differences in the host country, and lack of culturally appropriate services.

**Table 4:** Relevance of the emerging themes in the reviewed studies

S/*N*	Authors	Themes								
		Cultural challenges		Fathers’ needs and personal difficulties		Fatherhood in a new country				
		Cultural differences in the host country	Lack of culturally appropriate services	Information	Personal emotional and psychological challenges	Challenges and adaptation to fatherhood	Lack of extended family and building new support networks	Challenging traditional gender norms	Being a main supportive figure	
									Financial responsibility	Main supportive role during pregnancy, childbirth, and postpartum
1	Lee [32]	Y	Y	Y	Y	Y	Y	Y	Y	Y
2	Nges et al. [36]	Y		Y	Y	Y		Y		Y
3	Ny et al. [30]	Y	Y	Y	Y	Y	Y	Y	Y	Y
4	Valdez & Martinez [40]	Y		Y	Y	Y		Y		Y
5	Wojnar [31]	Y	Y	Y	Y		Y	Y		Y
6	Hunter-Adams [35]	Y			Y	Y	Y	Y		Y
7	Onyeze-Joe et al. [33]	Y		Y	Y	Y	Y	Y	Y	Y
8	Forbes et al. [37]	Y		Y	Y	Y	Y	Y	Y	Y
9	Riggs et al. [34]	Y	Y	Y	Y	Y	Y	Y	Y	Y
10	Capps et al. [38]	Y				Y	Y	Y		
11	Roubinov et al. [41]							Y	Y	Y
12	Khalil et al. [39]	Y			Y				Y	
13	Khalil et al. [43]	Y			Y				Y	
14	Khalil et al. [42]	Y			Y					Y

In general, the studies provide a brief description of the cultural barriers and need for culturally appropriate services. However, all of them were cross-sectional quantitative and qualitative studies and no study reported longitudinal research. Furthermore, the studies did not adequately capture how immigrant fathers navigate their cultural identity and adapt to new cultural norms.

#### Cultural Differences in the Host Country

The selected studies consistently highlight differences in cultural values and practices between the fathers’ home country and the host country. Ny et al. identified numerous cultural aspects with which migrant fathers and their families had to contend [30]. One of the significant challenges they encountered was language barriers. To access information and knowledge, they must learn the language of the host country. The differences also extend to beliefs shaped by the fathers’ original culture. For instance, one father expressed his desire to have “as many children as God is willing to give [31]”. However, that father felt troubled by the fact that Americans found this unusual, as they typically have no more than two children.

Differences also manifest in the healthcare field, as participants reported being unfamiliar with the perinatal healthcare system, such as undergoing mandated tests and how to work with the insurance system [32, 33]. Fathers also faced the challenge of reconciling traditional advice from their original country and that in the new country [30].

#### Lack of Culturally Appropriate Services

During the perinatal period, fathers have specific cultural needs not only for their partners but also for themselves. In 2012, Lee [32] found that Korean fathers in the United States desired culturally tailored healthcare services for their wives during the perinatal period This included services like Sanhujori-Won, a Korean postnatal care service providing comprehensive support for new mothers and their newborns [32]. They believed these services provided valuable professional and effective support for couples.

New fathers also discussed the need for a culturally tailored perinatal support program which provided appropriate information aligned with their cultural competence. Wojnar [31] highlighted the desire of Somali fathers in the United States for separate perinatal classes without explicit imagery. They stressed the importance of healthcare professionals displaying cultural sensitivity during labour and caring for their wives. Additionally, they faced challenges practising traditional customs, such as offering prayers for newborns, in their host country. Riggs [34] also identified that it is often believed to be inappropriate for men, even healthcare providers, to witness or touch women’s bodies. As a result, some fathers felt disrespected and frustrated when they did not have the option to choose the gender of the healthcare providers.

### Parenthood in a New Country

As depicted in Table 4, a prominent theme in the study explored the transition to fatherhood in a new country, examining various influencing factors. This theme encompasses four subthemes: Challenges and Adaptation to Fatherhood, Lack of Extended Family and Building New Support Networks, Challenging Traditional Gender Norms, and Being a Main Supportive Figure. Unfortunately, the data from the nine qualitative studies and five quantitative studies included in the review do not extensively delve into the fatherhood experiences of these fathers. Furthermore, none of the quantitative studies address the impact of lacking an extended family or assuming the role of the main supportive figure on the well-being of these fathers.

#### Challenges and Adaptation to Fatherhood

During the transition to fatherhood, migrant men underwent a significant transformation in their mindset, moving away from a self-centred perspective and embracing a more family-centred way of thinking. They changed their life goals as they adapted to their new role and prioritised the well-being of their partner and newborn baby [33]. Fathers embodied a metaphorical bridge between their original culture and the host country, striving to raise their children according to tradition in their new environment [30].

The role of fatherhood became apparent even before the child was born, as the husband showed concern for his wife’s health and expressed interest in the well-being of their unborn baby during prenatal check-ups [35]. The experience of witnessing the birth of their child was often regarded as the most profound and emotional moment of fatherhood. This moment provided fathers with a unique opportunity to connect deeply with the essence of fatherhood and reflect on their role in the family, particularly when they cut the umbilical cord for their infant [36]. At that moment, fathers experienced a deep connection with their wives and newborn children, who would now refer to them as “fathers” [37].

In general, eight qualitative studies and one quantitative study only offered basic descriptions of the common challenges and changes experienced by migrant fathers during the perinatal period. However, these studies did not delve into the transition process and the strategies fathers employ to adapt during this time.

#### Lack of Extended Family and Building New Support Networks

Eight qualitative studies and one quantitative study indicated that a lack of extended family is a common challenge faced by fathers during perinatal care. Typically, on migration to a new country, fathers leave behind extended family members who traditionally play a crucial role in providing support at this stage [33]. Fathers also shared that they did not have anyone to talk to about their difficulties [32]. Capp et al’s quantitative study [38] also demonstrated that in migrant families, additional adults from the extended family would often share the role of supporting the father during the perinatal stage.

Due to various reasons, not all migrant families have extended family members available to assist. The participants identified financial issues as the primary reason for the lack of extended family support. Hosting an extended family member was often challenging due to limited space in their small houses and the costs associated with supporting the extended family member’s arrival in the new country [35]. Moreover, due to travel expenses and the need to secure their financial status through employment, fathers and their families were often unable to visit their original country [32].

To compensate for the absence of extended family, fathers developed new support networks for themselves and their families, representing an opportunity for the fathers to form new relationships with other compatriots [37]. Additionally, migrant fathers sought support from local community resources, such as Korean churches, other immigrant families in similar age groups, and online social networks [32].

However, no quantitative study directly examined a hypothesis to test the role of extended family and new support networks for migrant fathers in their new country. Furthermore, there was a lack of studies testing the impact of the absence of extended family and new support networks on the well-being of these fathers.

#### Being a Main Supportive Figure

All of the selected studies provided data on the theme of being a main supportive figure. By synthesising the data, two subthemes emerged; financial responsibility and the main supportive role during pregnancy, childbirth and postpartum.

In general, the chosen studies provided a clear depiction of the primary support role of fathers. Nevertheless, none of these studies explored the long-term impact of assuming this role or delved into the coping strategies employed by fathers during the perinatal period.

**Financial Responsibility**. Due to societal gender norms and the unique health considerations of women during the perinatal stage, fathers often took on the role of breadwinners in the family [30]. The migrant fathers expressed that the primary reason for assuming this full responsibility is “*the sense of security in the family* [39]”. Additionally, these fathers believed that being a breadwinner was essential for them to serve as good role models for their children [30]. Some fathers also shouldered the responsibility of being financial providers for their family members back home [35].

**Main Supportive Role During Pregnancy**,** Childbirth**,** and Postpartum.** During the perinatal period, fathers played a vital role in providing support for every aspect of their partner’s experience. They took on logistical responsibilities such as driving to appointments, navigating the healthcare system, and assisting with interpretation during healthcare consultations [37]. They also fulfilled the supportive role of performing domestic chores to share the workload with their partner [33].

Although it may not be common in the participants’ countries of origin, migrant fathers were reported to assume the role of the primary supporter in the labour room for their partners. This experience has been described as *“opening up a new world for them* [30]*”*. The fathers experienced a range of emotions for themselves, but their primary focus was to provide mental support for their wives [36]. They also highlighted that being present in the labour room offered an opportunity to bond with their partners and the new baby [37].

#### Challenging Traditional Gender Norms and Roles

Eight qualitative studies and two quantitative studies provide data about the transition from traditional gender norms and roles. There were migrant fathers who persisted in adhering to rigid gender norms. They believed that household chores and caring for a baby were tasks primarily designated for women [36, 37]. In addition, the weight of financial responsibility extended beyond mere financial needs and was amplified by the pervasive influence of toxic masculinity, believing that as a father they needed to be the breadwinner to be a good role model to their child [30].

Nevertheless, there was a growing number of fathers who demonstrated understanding and flexibility regarding gender norms [40]. These fathers recognized the limitations of traditional norms and actively strove to adapt to new societal expectations within a new context. Lee [32] demonstrated that, unlike the traditional role of Korean husbands, Korean fathers residing in the US actively participate in perinatal care, including labour and infant care. They were also ready for cleaning, food shopping, and cooking. While certain cultures excluded men from the labour room, the host country prompted them to adapt and become primary supporters during childbirth, defying previous norms and fathers were pleased with that opportunity to support their wives [34].

Roubinov et al., [41] reported that, while fathers made efforts to be involved in perinatal care, they tended to avoid tasks traditionally associated with femininity such as bathing and diapering. Instead, fathers engaged in activities that correlate with positive aspects of masculinity, such as taking the baby out and playing with their baby.

In summary, while the qualitative studies provided valuable insights, no quantitative study formulated direct hypotheses regarding the topic of gender norms and the role of migrant fathers in their new country. Furthermore, all the data simply described the topic without delving into the process of this transition.

### Fathers’ Needs and Personal Difficulties

As shown in Table 4, nine qualitative and three quantitative studies highlighted the psychological distress, specifically stress and depression, experienced by fathers. Two subthemes were identified: Information, and Emotional and Psychological Challenges. The studies do not illustrate how these fathers cope with and recover from these challenges during the perinatal stage, indicating that additional research is required.

#### Information

Eight qualitative studies (see Table 4) mentioned the need for information and training, particularly in the context of culturally tailored programs. Fathers with migrant backgrounds often lack sufficient information and knowledge about the physical and psychological health of their perinatal partners, who undergo significant transitions during this period [40].

Fathers expressed a desire for more information to better prepare themselves for their role in the labour room [36, 37]. They also faced challenges in caring for the baby, as they felt unprepared and wished they had learned more beforehand. In addition, most fathers expressed that they had to acquire knowledge of perinatal care on their own [30, 33]. However, they found it inconvenient to access resources because these were not available in their native languages [31, 32].

However, the existing research predominantly relies on qualitative methods. There was a lack of quantitative studies with specific outcome measures to understand the impacts of lacking information and training. Consequently, we are unable to observe this phenomenon on a broader scale, nor can we explore its interaction with other variables, such as stress or the decision-making processes of fathers.

#### Emotional and Psychological Challenges

Nine qualitative studies and three quantitative studies showed that migrant fathers experienced a certain degree of psychological distress. According to Khalil et al. [39], approximately 35% of Arab American fathers met the diagnostic criteria for depression. A further study by Khalil et al. [42] discovered a significant reduction in telomere length among Arab American fathers, indicating a heightened level of stress among this group of men. These quantitative results show a consistent pattern with qualitative findings. Nges et al. [36] discovered that although Cameroonian fathers in Sweden experienced stress due to heavy workloads, they still made a significant effort to support their partners when they arrived home. These fathers expressed a loss of personal freedom and the burden of being the head of the household with increasing commitments and responsibilities [33]. In addition, they highlighted the stress from financial reasons as they were the breadwinner in their family [35, 43].

Some fathers openly mentioned their challenges when their wives faced mental health issues, particularly postpartum depression. During this time, fathers experienced a mix of emotions, often expressing feelings of helplessness and frustration [40]. In addition, fathers experienced high levels of stress while providing support in the labour room and witnessing the labour process, particularly when they witnessed pain or scenes with copious blood from their partners [32, 37]. Despite experiencing intense emotions, the fathers tried to suppress their feelings because of their role as main supporters [36, 37].

Among the three themes considered in this review, that of emotions and psychological challenges of migrant fathers stand out as the most prevalent in the selected studies. Nonetheless, none of the studies have explored the long-term impact of stress and negative emotions on these fathers. It might have been that those fathers would likely develop higher resilience as immigrants and would not have long-term effects from their perinatal experience. Either way, future research can investigate any such long-term effects. Furthermore, all the quantitative research has focused on Arab American fathers, indicating the need for further investigations into other populations.

## Discussion

This review provides a contemporary synthesis of studies on the lived experiences of migrant fathers during the perinatal period. The findings underscore the significance of cultural challenges, the transition to fatherhood in a new country, and the personal needs and difficulties of the fathers. Building on these findings, this discussion is structured into two sections. The first section delves into the depth of the cultural challenges by examining how the fathers negotiate gender norms and core cultural values. The second section addresses their transition to fatherhood including their needs and personal difficulties.

### Negotiating Gender Norms and Core Cultural Values

The selected studies showed that the migrant fathers share caring roles and childcare responsibilities with their partners. This aligns with the findings of Mprah et al. [8], which highlight cultural clashes experienced by migrant fathers navigating differing fatherhood expectations between their home country and the host country. However, this current review provides more nuanced insights into these cultural clashes among a larger cohort of migrant fathers. The selected studies in this review showed that in certain cultures, like Chinese, Korean, and Somalian, involvement in pregnancy, childbirth, and postpartum care might be viewed as breaking traditional gender norms [31, 32, 44]. Therefore, as migrant fathers embrace their new roles in a host country, they change their male identities and family gender roles. Gender identity is a crucial aspect of culture and gendered processes play a major role in the acculturation process [45]. Thus, negotiating gender norms has emerged as a prominent theme during acculturation [46]. This necessitates a flexible approach requiring migrant fathers to redefine gender roles within the home and become comfortable with tasks that are traditionally perceived as feminine in their country of origin.

The involvement of migrant fathers during the perinatal stage is crucial due to a lack of support from their extended family in the host country. This necessary participation provides fathers with opportunities to break traditional norms, positively impacting their ability to support their partners. Some fathers express positive views on cultural and traditional changes, suggesting a potentially less stressful perinatal period. Understanding how migrant fathers perceive the opportunities versus the costs of breaking traditional norms is essential. Moreover, traditional norms indirectly affect migrant fathers, as seen in Ethiopia, where cultural stereotypes portraying men as unhelpful in childcare impact fathers’ self-perceptions, influenced by perpetuation in their home country and host country practices [37]. Health professionals and mothers in the host country often afford fathers more space for caregiving responsibilities compared to the constraints imposed by cultural stereotypes in their country of origin [47].

The current review also shows that many migrant fathers maintain their heritage cultural values while attempting to adopt the host country’s values. Unlike integration, an acculturative strategy in which the individuals embrace both sets of cultural values [48], migrant men in the perinatal period found themselves at the intersection of their original cultural values and the host culture, leading to a complex negotiation process that was more complicated than simply being fathers in their home country or acculturating immigrants. This process involves negotiating cultural practices to align with the new culture. The adaptation process varies as fathers selectively adopt cultural practices, modify traditional gender roles, and embrace new gender identities in their new contexts. Achieving this is challenging; however, it is evident that successful integration into the new society facilitates a faster acculturation process [48] and reduces the risk of mental health illness [49].

### Transition to Fatherhood in a New Country

The studies included in this review provide valuable insights into the experiences of immigrant fathers as they navigate the complexities of fatherhood in a new country. The emergent themes in this review show that this process in a new country is closely intertwined with the negotiation of gender norms and core cultural values. During the transition, fathers develop a sense of being a parent to their newborn child while aiming to keep a balance between their cultural values and the new values of the host country. This tension requires fathers to make decisions about which values and customs to preserve for their family and the upbringing of their children. This leads to a renegotiation of their roles as they navigate the demands of their cultural heritage alongside the expectations of the new environment.

In this review, the findings also show the needs and personal difficulties of the fathers. This aligns with previous research by Mprah et al. demonstrating that, like fathers in general, migrant fathers must navigate their evolving roles, including caring for their partner and new baby, adjusting attitudes and behaviours, and confronting various challenges along the way [8]. However, the current review further reveals that the transition for migrant fathers is particularly challenging due to the simultaneous pressures of acculturation and a multitude of other factors. In addition to navigating the complexities of adapting to a new culture, these fathers must contend with additional stressors such as the absence of extended family [38], financial constraints [50], inadequate parenting skills [12], and challenges in managing stress [7]. These various factors combine to intensify the difficulties faced by these migrant fathers during this transformative period and resonated across all the reviewed studies. This emphasizes the crucial need for support systems tailored to the migrant fathers during this transformative period.

The examined studies also emphasize the significance of supporting migrant fathers through societal structures and healthcare systems during their transition to fatherhood. Host countries typically provide assistance such as parental leave policies, healthcare services, and culturally tailored perinatal support programs [51]. However, challenges arise for international students or those on temporary working visas, especially in countries like Australia, New Zealand, and the United States, where eligibility for government support and parental leave is often restricted due to casual employment [52, 53]. This limitation can hinder fathers in fulfilling their roles, particularly when they become the primary breadwinners for their families. In addition to government support, social support from networks and communities is crucial for migrant fathers during this transition. These supportive relationships offer emotional, and practical assistance and role models to help fathers adjust and fulfil their new roles [54]. Such support becomes especially valuable for migrant fathers who lack the presence of extended family in their new country, offering opportunities to redefine gender roles when facing challenges like the mother’s early return to work or the necessity for long working hours to support the family [55].

Despite the many challenges facing migrant fathers, these studies also illuminated the resilience of those fathers. Regardless of the difficulties, all fathers reported feeling a strong sense of fulfilment in their role while raising the baby and taking care of their partner [30, 32, 33]. Furthermore, these fathers, driven by their commitment to family, consistently strive to acquire knowledge and skills to support themselves as their families go through the challenges in the period. Also, this resilience could motivate these fathers to reach out to their community for support and develop helpful connections in the new country [56].

### Strengths and Limitations

This review combines qualitative and quantitative studies for a comprehensive analysis of migrant fathers’ perinatal experiences. However, due to scope limitations, it does not include grey literature or articles in languages other than English. Besides, all the included studies were conducted in developed countries, and most of them focused on migrant fathers from developing countries. Therefore, the review may not capture all aspects across diverse backgrounds. Also, since research found that reasons for migration influenced outcomes [57, 58], the current review kept the search terms for the participants as broad as Migrant, hoping to capture studies on its sub-categories (e.g., refugee and asylum seekers). However, none of the selected studies specifically detailed the reasons for migration among their participants. Therefore, the findings in the current review could not differentiate between the experiences of forced migrants and non-forced migrants. Finally, the heterogeneity in methodology is both a strength and challenge, hindering conclusive common themes. Future research could explore homogenous groups, focusing on migrant fathers with the same origin and host country for a more nuanced understanding.

## Conclusion

This review synthesizes recent studies on migrant fathers’ experiences during the perinatal period and it addresses significant gaps in existing literature that predominantly focuses on mothers. The findings underscore the importance of redefining traditional masculinity and increasing support for migrant fathers. While some community efforts exist, they lack adequate research attention to establish their efficacy. Encouraging more collaborations between perinatal researchers and community service providers is essential for developing and evaluating support programs for migrant fathers. This not only benefits fathers and their families but also contributes to the education and training of future clinicians. In conclusion, the review identifies common themes in migrant fathers’ experiences, emphasizing the need for further research and clinical interventions to address these issues and bridge existing gaps.

## Electronic supplementary material

Below is the link to the electronic supplementary material.

Supplementary Material 1

Supplementary Material 2
