# Supplementary material for: Lived Experiences of Migrant Fathers in the Perinatal Period: A Systematic Review and Analysis
Source: J Immigr Minor Health. 2024 Aug 29;26(6):1070–84. doi: 10.1007/s10903-024-01627-0 (PMC11607057; doi:10.1007/s10903-024-01627-0)
Supplement: Supplementary file 1 — Supplementary Material 1 [file 10903_2024_1627_MOESM1_ESM.docx]

|  |  |  | | | | | | | | |
| --- | --- | --- | --- | --- | --- | --- | --- | --- | --- | --- |
| **Category of study designs** | **Methodological quality criteria** | **Lee (2012)** | **Nges et al (2022)** | **Ny et al (2008)** | **Valdez & Martinez (2019)** | **Wojnar (2015)** | **Hunter-Adams (2016)** | **Onyeze-Joe et al (2022)** | **Forbes et al (2021)** | **Riggs et al (2016),** |
| Screening questions  (for all types) | S1. Are there clear research questions? | Y | Y | Y | Y | Y | Y | Y | Y | Y |
|  | S2. Do the collected data allow to address the research questions? | Y | Y | Y | Y | Y | Y | Y | Y | Y |
| 1. Qualitative studies | 1.1. Is the qualitative approach appropriate to answer the research question? | Y | Y | Y | Y | Y | Y | Y | Y | Y |
|  | 1.2. Are the qualitative data collection methods adequate to address the research question? | Y | Y | Y | Y | Y | Y | Y | Y | Y |
|  | 1.3. Are the findings adequately derived from the data? | Y | Y | Y | Y | Y | Y | Y | Y | Y |
|  | 1.4. Is the interpretation of results sufficiently substantiated by data? | Y | Y | Y | Y | Y | Y | Y | Y | Y |
|  | 1.5. Is there coherence between qualitative data sources, collection, analysis and interpretation? | Y | Y | Y | Y | Y | Y | Y | Y | Y |
| Y=Yes, N=No, CT=Can’t tell | | | | | | | | | | |

**Supplementary table S1.** MMAT ratings for qualitative, quantitative, and mixed method studies

| **Category of study designs** | **Methodological quality criteria** | **Capps et al (2010)** | **Roubinov et al (2016)** | **Khalil et al (2022a)** | **Khalil et al (2022b)** | **Khalil et al (2023)** |
| --- | --- | --- | --- | --- | --- | --- |
| Screening questions  (for all types) | S1. Are there clear research questions? | Y | Y | Y | Y | Y |
|  | S2. Do the collected data allow to address the research questions? | Y | Y | Y | Y | Y |
| 2. Quantitative descriptive studies | 2.1. Is the sampling strategy relevant to address the research question? | Y | Y | Y | Y | Y |
|  | 2.2. Is the sample representative of the target population? | Y | Y | CT | CT | CT |
|  | 2.3. Are the measurements appropriate? | Y | Y | Y | Y | Y |
|  | 2.4. Is the risk of nonresponse bias low? | Y | Y | CT | CT | CT |
|  | 2.5. Is the statistical analysis appropriate to answer the research question? | Y | Y | Y | Y | Y |
