# Supplementary material for: Lived Experiences of Migrant Fathers in the Perinatal Period: A Systematic Review and Analysis
Source: J Immigr Minor Health. 2024 Aug 29;26(6):1070–84. doi: 10.1007/s10903-024-01627-0 (PMC11607057; doi:10.1007/s10903-024-01627-0)
Supplement: Supplementary file 2 — Supplementary Material 2 [file 10903_2024_1627_MOESM2_ESM.docx]

**CAPTION: A summary table of themes and data extracts**

**Online supplementary material for Lived Experiences of CALD Fathers in the Perinatal Period:
A Systematic Review and Analysis**

*Table of first, second, and third order themes with example data extracts*

**First Order Theme: Cultural challenges**

| **Second order theme** | **Third order theme** | **Data codes** |
| --- | --- | --- |
| **Cultural differences in the host country** |  | “Fathers reported they saw changing their behavior and taking a larger role in the family not only as helpful for their partners but also as a way to challenge themselves and their gender role beliefs and to increase understanding and ﬂexibility” (Valdez & Martinez, 2019, p. 314) *  “Traditionally, husbands were not allowed to be present during their wives’ labor in Korea. Findings in this study suggest that all of the participating husbands felt that they were expected to take an active role during the labor in the U.S. health culture” (Lee, 2012, p. 124) *  “Oh, especially, the ultrasound tests. During our first and second pregnancy, we could have only one ultrasound test because our insurance covered it only one time. We knew in Korea, you can have an ultrasound test whenever you go to doctor’s office if you want. But here, one time, and that’s it. So, I became so nervous especially at the end of pregnancy because I wanted to see my baby with my eyes and make sure (s)he was growing well inside. But when I asked [health care providers], they said I had to pay everything out of my pocket for that. And it was, wow, too expensive for us. It is so different from Korea and we really did not like it at all” (Lee, 2012, p. 148) *  “You see, even if, like, she has a church where she’s a part of a church, you’ll see they will give even the announcements to the church—“Look, we’ve got our sister, she has a baby now, we have to go and visiting her, so we can’t go hands empty, so we have to bring something to her,” so they will just collect something for her, so they will bring something for her and you’ll see all, even the whole month I can say, people got enough time because they’re doing nothing, they can come, someone can come stay there from morning till late, even sleep there, spend even 2 days or 3 days, even a week, because they’ve got enough time” (Hunter-Adams, 2016, p. 1016) *  “The reciprocal and extensive set of physical, cognitive, and emotional supports had real-time costs, making it difﬁcult for migrants to reestablish these links in a new country, particularly given the continued ﬁnancial commitments to family at home” (Hunter-Adams, 2016, p. 1021) *  “We found that maternal and paternal acculturative stress, anxiety, and depressive  symptoms were significantly correlated” (Khalil, 2022b, p. 5)  “It felt like adapting was the only way to become the father their new environment and partners expected them to be” (Onyeze-Joe et al., 2022, p. 5) *  “I knew there would be differences if I change from my home country to another country . . . if I have to survive, I have to adapt to the new way of life . . . Now in our home, I cook, back home I never cooked” (Onyeze-Joe et al., 2022, p. 5) *  “The clash of cultures refers to the struggle the fathers faced with the western practice of fatherhood in Belgium that were contrary to their own conceptions of fatherhood” (Onyeze-Joe et al., 2022, p. 5)  “From my own experience with fathers, I knew back home, none of them said they were going to the hospital with their wives for checkups or other things, but they are always supporting, like buying vegetables, buying fruits and providing money for the needs of the family. But being physically present, I don’t think that’s the case. On the day of the delivery, many of them were at work or somewhere else when they were told that their wives had delivered” (Onyeze-Joe et al., 2022, p. 5)  “For some people, the issue of circumcision is an issue of religion. In my case, it’s more cultural, than religious. It’s what we do, culturally. Even though I share a similar religion with my spouse, and so it should be easy to decide about circumcision, but my son is not circumcised yet. Because Belgians don’t believe in circumcision and the hospitals are not yet equipped to handle that, I have had to look at it logically and see if it matters. We are both Catholics and here it’s not a priority for Catholics in Belgium. I have had to look at the logic of it and I am not convinced in myself to continue in that culture if it is not ok with her. It doesn’t make sense to force her to accept it” (Onyeze-Joe et al., 2022, p. 6)  “The fathers shared decision-making with their spouses is an indication of acculturation motivated by a quest to fit into the host culture and to possibly gain acceptance” (Onyeze-Joe et al., 2022, p. 7)  “All the men stated that knowledge of Swedish is important when taking part in group education, as this is mostly offered in Swedish” (Ny et al, 2008, p. 284)  “Advice given by the maternity and child-health care services in Sweden was compared with the experiences and advice given back home” (Ny et al, 2008, p. 284)  “The men stated that old and new advice were often used in conjuction with older traditions. Some advice was considered strange, but, after talking to the midwife or child health-care nurse, the advice was often accepted” (Ny et al, 2008, p. 284)  “I see in this society that I have to go with my daughter son or wife, side by side, I must know” (Ny et al, 2008, p. 285) *  “I believe that there is a huge difference, from which country you come from, to which culture you belong to, and also it differs within the same country and the different classes in the community, coming from the countryside or the city” (Ny et al, 2008, p. 285)  “Becoming integrated can be difﬁcult because of structural social problems, such as isolation within segregated areas, crowded housing and no opportunites for employment” (Ny et al, 2008, p. 288) *  “It was a major step to take for the Cameroonian men to accompany their women into the maternity unit, an unfamiliar area” (Nges et al., 2022, p. 3) *  “All of the couples expressed a desire to have “as many children as God is willing to give” and felt troubled that so many American couples had one to two children or no children at all” (Wojnar, 2015, p. 362) *  “By weighing medical advice against their own life experiences and the opinions of healers and elders in the Somali community, the women and men felt they could navigate the health care system easier. For example, the majority of study participants believed that routine pregnancy tests and dietary adjustments are not necessary because pregnancy is a “normal” state” (Wojnar, 2015, p. 363)  “When our last son was born, I went to hospital with her because there were no women to go with her. I knew some English but my she didn’t and we had a computer translation. But, when the baby came out, they turned it off. They took my baby away and gave him shots. I thought something was wrong with him and I started crying. When the nurse saw it she asked: “Are you crying because you are happy?” and I “No, what is wrong with my baby?” and she said “Nothing, he is getting normal care.” I wanted to hold the baby ﬁrst and say a blessing like I did back home but I didn’t know how to ask or if I could. I wish I had the opportunity to do it my way. I just want them to accept and respect us just as we are, but I didn’t know how to say it...” (Wojnar, 2015, p. 363 - 364) *  “Male participants tended to be less concerned about their wife getting the “right” episiotomy and more concerned about them not having the cesarean birth. They consistently indicated that the memories of “women all the time dying in surgery back home” was one of the key reasons they opposed cesarean birth. Some also expressed concerns that cesarean section would limit the number of children they wanted” (Wojnar, 2015, p. 364) *  “The longer you are in this country, the smarter you become about the differences between you and the Americans and about your rights. You begin to understand that you have to be your own advocate in health care because nobody else will. It is very true when you go to hospital to have a baby. If you don’t ask the right questions they will assume you understand and agree to everything while you don’t” (Wojnar, 2015, p. 364) *  “Social support, especially from those who are themselves Americanized, may encourage the use of parenting practices that conflict with traditional notions of parenting; and support may also reinforce traditional beliefs of parenting practices” (Capps et al., 2010, p. 85)  “Paternal acculturative stress had a signiﬁcant moderate positive correlation with mothers’ PPD symptoms (r = .39, p = .04)” (Khalil et al, 2022a, p. 96) *  “Maternal and paternal acculturative stress scores were signiﬁcantly correlated (r = .61, p < .001)” (Khalil et al, 2022a, p. 96) *  “Men reported that in Afghanistan it would be considered dishonorable or shameful for a man to attend his wife’s delivery, but in Australia men’s involvement was necessary and expected” (Riggs et al., 2016, p. 88) *  “Being involved in pregnancy care challenged traditional values and beliefs” (Riggs et al., 2016, p. 88) *  “You go inside the maternity room and you see when the baby comes out. That’s not in back home, they’d be coming out only the midwives would go with them….. [Here] they allow me to cut the umbilical cord and cut with the scissors. Yep It’s good and it’s a bit painful to see all that drama” (Forbes et al., 2021, p. 5) * |
| **Lack of culturally appropriate services** |  | “All participating couples identified having a limited support system as a major challenge. The couples stated they were in great need of extra support, especially during the Sanhujori period” (Lee, 2012, p. 118)  “This subtheme refers to the perceived challenge associated with the absence of a Korean traditional postpartum care center, a Sanhujori-Won, in the local community” (Lee, 2012, p. 122)  “I heard good things about Sanhujori-Won when I was visiting Korea. I also heard about it from other [Korean] people here in the U.S. It sounds like a mom can recover much better and faster in Sanhujori-Won because everything would be taken care of by them [Sanhujori-Won staff]” (Lee, 2012, p. 123)  “And then, the doctor came in and introduced himself as the on-call doctor for the day. He seemed very nice, so we decided not to insist getting our doctor any more at that time. ... And we expected our doctor to say a word something like ’I’m sorry [for not being there for your labor] when we went to see her for her [postpartum] check-up [in English]. But she [the doctor] did not say anything. So, we were so disappointed ... but it seemed like she did not know that we considered her as our Joo-Chi-Eui. Thus, when I got pregnant again, I switched to doctor [the Korean immigrant doctor] because I heard that he does everything by himself, even delivery, [brief pause] That is why” (Lee, 2012, p. 145 - 146)  “They felt somewhat ignored when their U.S. health care providers were not willing to give them advice on things for which they asked their opinion” (Lee, 2012, p. 146)  “The stories from the couples who took prenatal classes (n = 5) or who wanted to take ones but could not (n — 9) suggested that they desired the prenatal classes here to be culturally tailored to better fit what Korean immigrant couples wanted to know” (Lee, 2012, p. 152)  “Several men stated that a good father is a person who can combine the two worlds, bringing up the children as tradition calls for but in a new country” (Ny et al., 2006, p. 285)  “All of the couples expressed a desire to have “as many children as God is willing to give” and felt troubled that so many American couples had one to two children or no children at all” (Wojnar., 2015, p. 362) *  “They hoped the caregivers would somehow know when to provide them with information without making assumptions. Because the participants often didn’t understand what was going on, they missed opportunities that were important to them for religious and cultural reasons” (Wojnar., 2015, p. 363) *  “No, what is wrong with my baby?” and she said “Nothing, he is getting normal care.” I wanted to hold the baby ﬁrst and say a blessing like I did back home but I didn’t know how to ask or if I could. I wish I had the opportunity to do it my way. I just want them to accept and respect us just as we are, but I didn’t know how to say it...” (Wojnar., 2015, p. 364) *  “Some participants felt that because they had different expectations from pregnancy and childbirth than the Americans, the providers broke their promises or disregarded wishes. Such situations led the study participants to feeling disrespected, lonely, and longing for a better relationship with health care professionals. Similar to others, one man recalled” (Wojnar., 2015, p. 364)  “My wife’s doctor mentioned prenatal classes but we didn’t attend after we Checked them out. Our religion doesn’t allow us to attend classes with the women learning in the same room as men. And, we are not allowed to look at naked ﬁgures and so on. I think many Somalian men would definitely go if the setting was respectful of our beliefs. I know I would go to better advocate for my wife. Doctors and nurses think we don’t care but we do. For example, after we arrived in the hospital to have the last baby I was asked to complete some forms. Under the prenatal classes I checked “none.” The nurse remarked: “not interested, huh?” Well, I was, but I felt it wasn’t a good time to educate the nurse why I didn’t go. And so, I didn’t” (Wojnar., 2015, p. 365)  “My wife had a cut (FGC) as a child; a very bad cut. After she had the last baby in the US, the doctor took me aside, explained how bad it was and suggested we should consider having it repaired. He didn’t pressure me, just had a conversation. I was open to the idea because my wife had health problems because of it but my she was against it at ﬁrst. We talked about it more after coming home and then went back to see that doctor in strictest conﬁdence. Nobody knows she had the surgery, only me. It is the most taboo topic in our culture” (Wojnar., 2015, p. 366)  “Culturally appropriate prenatal programs and individual supportive interventions offered to Somali couples in the perinatal period may help more women to seek reconstructive surgery” (Wojnar., 2015, p. 367)  “For men, trust was an important and positive element in their relationships and interactions with care providers. Everything is good in pregnancy care because we trust them” (Riggs et al., 2015, p. 89)  “The only disrespect was that we couldn’t choose the doctor to be female during labor. My wife was uncomfortable and worried. They [health professionals] said it’s no issue for us and shouldn’t be for you guys” (Riggs et al., 2015, p. 89) |
| * extract coded to more than one theme | | |

**First Order Theme: Parenthood in a New Country**

| **Second order theme** | **Third order theme** | **Data codes** |
| --- | --- | --- |
| **Challenges and Adaption to Fatherhood** |  | “For fathers, truly supporting their partner meant recognizing and changing their own behavior (i.e., drinking with friends). It also meant increasing their presence at home” (Valdez & Martinez, 2019, p. 313)  “Fathers reported they saw changing their behavior and taking a larger role in the family not only as helpful for their partners but also as a way to challenge themselves and their child” (Valdez & Martinez, 2019, p. 314) *  “The fathers recognized the need to increase involvement with their children” (Valdez & Martinez, 2019, p. 314)  “Fathers said they countered the helplessness they felt with their partners’ depression by focusing on their children, which gave them a sense of mastery and enjoyment” (Valdez & Martinez, 2019, p. 315)  “The fathers in our case study seemed to defy gender stereotypes of traditional machismo and to embrace caballerismo with increased closeness and involvement with their children, as found in our themes of family support and adaptive coping” (Valdez & Martinez, 2019, p. 318)  “Some fathers described the desire to be “good fathers,” which they contrasted with their own fathers’ failures because of alcoholism, inﬁdelity, and abandonment” (Valdez & Martinez, 2019, p. 318)  “Even when I cut the cord, there was blood oozing out from it. That was a little bit... and uh... but at that time though, because I was too excited to think of anything else but we just had a baby. So I did not feel much anything about it [blood]. But later on, when I recalled the labor room, oh ... my, my. I think it was not easy to deal with the bloody scene” (Lee, 2012, p. 124)  “When they could ﬁnd a car to use and ﬁnancial provider, all while having to take an active role in raising their new baby” (Hunter-Adams, 2016, p. 1018)  “Some men did discuss the health of the baby separately from the health of their partners, and they felt a duty to both their partner and their unborn child” (Hunter-Adams, 2016, p. 1018)  “Men described having a much closer bond and knowing details of their children’s lives they would not have known previously in their home country” (Forbes et al., 2021, p. 5)  “You go inside the maternity room and you see when the baby comes out. That’s not in back home, they’d be coming out only the midwives would go with them….. [Here] they allow me to cut the umbilical cord and cut with the scissors. Yep It’s good and it’s a bit painful to see all that drama” (Forbes et al., 2021, p. 5) *  “Men described the responsibility of being the breadwinner and also attempting to take care of their partners and children physically and emotionally through the perinatal period, as challenging and stressful” (Forbes et al., 2021, p. 8) *  “You are responsible for everything from pregnancy and childbirth and then raising children. So it would be very, very, very difficult for a single person” (Forbes et al., 2021, p. 8) *  “Men should be involved to help them understand the suffering and challenge involved in pregnancy, birth and caring for children” (Forbes et al., 2021, p. 8) *  “Participants also said it was important for men to be involved in the perinatal period overall, to support fathers’ bonding and relationship with their children” (Forbes et al., 2021, p. 9) *  “Participants suggested that including men supported the couple’s relationship and the father’s bond with their child, allowed men to understand the processes involved with pregnancy, childbirth and infant-care and improve their health literacy” (Forbes et al., 2021, p. 11) *  “The desire of all the participants to become fathers was apparent in their narratives. As one participant put it, “I was very expectant to have a child”” (Onyeze-Joe et al., 2022, p. 4)  “They spoke about the loss of personal freedom to the increasing daily commitments and responsibilities during the pregnancy and then as fathers” (Onyeze-Joe et al., 2022, p. 4)  “I wasn’t doing my regular fun activities like watching football, doing the things I loved to do. All I did was what I could do at that moment. I was there for my wife and the baby. Before then, I went to play football like twice a week, I could not do that anymore. All my fun activities just ended with my new reality” (Onyeze-Joe et al., 2022, p. 5) *  “It felt like adapting was the only way to become the father their new environment and partners expected them to be” (Onyeze-Joe et al., 2022, p. 5) *  “The clash of cultures refers to the struggle the fathers faced with the western practice of fatherhood in Belgium that were contrary to their own conceptions of fatherhood” (Onyeze-Joe et al., 2022, p. 5) *  “The narratives of the first time fathers revealed changes in their identity and a shift in their priorities as new fathers. Many spoke about changing life goals and priorities” (Onyeze-Joe et al., 2022, p. 6)  “Beyond the new responsibilities the men embraced in the home, the fathers spoke about scheduling work around their family needs” (Onyeze-Joe et al., 2022, p. 6)  “One informant described this phase as a time of evolution from a “me” mentality to a “us” mind-set. Notion of changing mindsets was portrayed as a change in lifestyle from seeking personal goals to being more family-oriented” (Onyeze-Joe et al., 2022, p. 6 - 7)  “Fatherhood has taught me a lot . . . I have learned to pull myself together, to re-evaluate my choices to adapt to my new life. This has made me cultivate a kind of patience and a kind of introspection about how I function as a father” (Onyeze-Joe et al., 2022, p. 7)  “Although challenging at first, many of the fathers were able to adjust to their new role with time and even prioritized their family time over other personal needs” (Onyeze-Joe et al., 2022, p. 7)  “I see in this society that I have to go with my daughter son or wife, side by side, I must know” (Ny et al., 2008, p. 285) *  “Several men stated that a good father is a person who can combine the two worlds, bringing up the children as tradition calls for but in a new country” (Ny et al., 2008, p. 285) *  “Here you are an extra weight to your wife. If you have money and work, you are going to teach your son to work also. You become a role model to your son” (Ny et al., 2008, p. 286) *  “The men also discussed how difﬁcult it was to be a role model for your children when you had no job and no status” (Ny et al., 2008, p. 288) *  “Fathers stated that being there as the closest person to their women and seeing her undergo severe and enduring pain was traumatizing and brought feelings of frustration. However, being there to comfort and share the hardship with the woman gave a sense of meaningfulness to the fathers” (Nges et al., 2022, p. 4) *  “Feelings such as happiness in holding the baby and hearing the baby’s cry for the ﬁrst time, closeness to the woman, and thankfulness that the outcome was successful” (Nges et al., 2022, p. 4) *  “Fathers found themselves bonding naturally with their women and babies. Most importantly, they felt more connected to their families and nothing else mattered at that point, just their babies and their women” (Nges et al., 2022, p. 5)  “The birth process uncovered new emotions which brought about a sense of higher self-awareness. The experiences as reported, gave existential lessons about the way of life as well as the core values of one’s own life” ” (Nges et al., 2022, p. 5)  “That is a special feeling, (to cut the umbilical cord) you know that haha. It is a special feeling for all men. Just ask all the men who have done it. It is very special for you to be there, I mean do that. I felt like a man, yes, I felt manly. That is when I became a full man as I can say it even. Because I was a half man, so I became full haha. Its a big deal, yeah. It is good” (Nges et al., 2022, p. 5) *  “Social support, especially from those who are themselves Americanized, may encourage the use of parenting practices that conflict with traditional notions of parenting; and support may also reinforce traditional beliefs of parenting practices” (Capps et al., 2010, p. 85)  “Having ﬁnancial security to support their wives, newborn baby, and other children was important to all of the men. Yet employment also proved to be a dilemma for men who were anxious about their availability to support their wives” (Riggs et al., 2016, p. 88)  “I was happy. I wanted to help her, because being a father has responsibilities to take care of them, my culture tells me to take care of my children and family” (Riggs et al., 2016, p. 89) * |
| **Challenging traditional gender norms** |  | “Fathers who reported they felt stigmatized when they talked about depression in Mexican culture” (Valdez & Martinez, 2019, p. 312)  “With an increased presence at home, Leo became more involved in household chores. “Sometimes I do laundry or I clean the house” (Valdez & Martinez, 2019, p. 313)  “Fathers seemed to increase involvement in the home not only to support the mother but also to enhance marital exchanges” (Valdez & Martinez, 2019, p. 313) *  “The fathers conveyed that their previous expectations for the mothers to fulﬁll most household and caregiving roles were harmful to their partners’ wellbeing and rooted in traditional gender norms of machismo” (Valdez & Martinez, 2019, p. 314)  “The fathers noted that men in their lives (e.g., fathers and brothers) ascribed to traditional machismo beliefs and saw fulﬁlling household chores as being “bossed around” by their wives” (Valdez & Martinez, 2019, p. 314) *  “Fathers reported they saw changing their behavior and taking a larger role in the family not only as helpful for their partners but also as a way to challenge themselves” (Valdez & Martinez, 2019, p. 314) *  “Fathers reported they saw changing their behavior and taking a larger role in the family not only as helpful for their partners but also as a way to challenge themselves and their gender role beliefs and to increase understanding and ﬂexibility” (Valdez & Martinez, 2019, p. 314) *  “Culture (machismo, stigma) and social context (relationships, mental health and substance use history, poverty) inﬂuence and are enacted in fathers’ experiences and their emotional response” (Valdez & Martinez, 2019, p. 317)  “Fathers in our case studies indicated that they changed their behavior and roles in tandem with their better understanding of maternal depression. They concurred that receiving accurate information about maternal depression was key to understanding it, which, in turn, allowed them to manage their emotional responses and to overcome constraints of traditional machismo to support the family” (Valdez & Martinez, 2019, p. 317) *  “The fathers in our case study seemed to defy gender stereotypes of traditional machismo and to embrace caballerismo with increased closeness and involvement with their children, as found in our themes of family support and adaptive coping” (Valdez & Martinez, 2019, p. 318)  “Our study extends models of Latinx caregiving by highlighting how fathers defy traditional gender norms to become caregivers when mothers experience depression and withdraw from the family” (Valdez & Martinez, 2019, p. 318)  “The challenges experienced by Korean immigrant husbands associated with taking non-traditional gender roles during labor and helping their wives with childcare and housework” (Lee, 2012, p. 124)  “Unlike the traditional role expectations for Korean husbands, all of these husbands stated that they were involved in perinatal care, including the labor process and infant care (e.g., diaper changing, bathing the infant), as well as housework such as cleaning, grocery shopping, and cooking” (Lee, 2012, p. 125)  “Sometimes, I feel it is hard [to do housework]. We cannot afford to hire a housekeeper. I have to do it [housework], otherwise my wife has to do all the work” (Lee, 2012, p. 126) *  “Moreover, when we decided to have a family and a baby here, I felt so much pressure on my shoulders ... but I should be in charge and do my best. I am the head of household here ... Everyone depends on me. I don’t know whether it is because I am simply getting old or because I am being more mature. But I just think about the responsibility [as the head of household] a lot” (Lee, 2012, p. 129) *  “All husbands considered their experience as an active partner or a labor coach to be a life-changing one” (Lee, 2012, p. 130) *  “It’s quite a big deal when it comes to being a husband.…It’s quite scary.…Your wife is pregnant now, then from that moment on it becomes, it remains your responsibility, according to our culture, in terms of helping the wife” (Hunter-Adams, 2016, p. 1018) *  “While circumstances and some gender roles— what men and women actually did day to day—had changed for migrant men and women in their move to South Africa, men did not talk about the women with whom they had relationships as fully autonomous or capable” (Hunter-Adams, 2016, p. 1018)  “You know, a pregnant woman, she’s like a small child, a baby, that’s what is a pregnant woman, that’s the example that I can give to a pregnant woman: a child. If you’ve got a child, you are the one who choose the food for her. Sometimes she don’t want that food and you don’t know what she want, so same applies to your woman” (Hunter-Adams, 2016, p. 1019)  “It was described around some father’s reluctance to engage with young babies, because they were not comfortable, or some families still living in traditional ways” (Forbes et al., 2021, p. 8)  “They’re not interested in involving the maternal.. Yeah even here. There are some men they don’t hold a newborn baby until two 3 months” (Forbes et al., 2021, p. 8)    “At least one man and several women said that not all Ethiopian men were actively engaged in the perinatal period or in domestic tasks involved with raising children” (Forbes et al., 2021, p. 9)  “I knew there would be differences if I change from my home country to another country . . . if I have to survive, I have to adapt to the new way of life . . . Now in our home, I cook, back home I never cooked” (Onyeze-Joe et al., 2022, p. 5) *  “Since the very first appointment with the doctor, I had always been present, which is not customary where I come from . . . Back home, as a man, if your wife is pregnant or your wife delivers nothing changes, you just continue with your life. It’s like you get a phone call, your wife has delivered, you are so happy, you tell your friends your wife is at the hospital, she has just given birth . . . nothing changes. Because usually, her mother is there with her and the husband has no role to play there” (Onyeze-Joe et al., 2022, p. 5) *  “The fathers acknowledged that being the man did not exempt them from domestic responsibilities, as is the case back home. Instead, fathers in Belgium were expected to share parental and domestic duties equally” (Onyeze-Joe et al., 2022, p. 5) *  “Sometimes you, as the man, have to reschedule things to assist. I work in shifts. If I have the morning shift, then she can feed the baby at night and vice versa. We support each other like that. She doesn’t do everything like the housework. We all shuffle it out” (Onyeze-Joe et al., 2022, p. 6)  “The fathers acknowledged being coplayers with their partners at each stage from pregnancy, birth, and in child care. Even though they strongly believed in their role as breadwinners, they were willing to also embrace domestic and child care responsibilities as well” (Onyeze-Joe et al., 2022, p. 7) *  “The African fathers in our study agreed that a father’s most important role is to provide in African cultures. Hence, it is expected that the “other support” is filled by extended female family members, especially at the critical moment of birth and early child care” (Onyeze-Joe et al., 2022, p. 8) *  “In their native countries, it was the female network, with the mother-in- law in charge, that helped and took care of women during pregnancy and childbirth: In Sweden the man has taken her place” (Ny et al., 2008, p. 285) *  “One man in group (B) did not want to join his wife during the birth because ‘it was not a place for men’. His wife was to be accompanied by a female relative or friend and, according to the man, this was something that they had agreed upon” (Ny et al., 2008, p. 285) *  “They acknowledged that childbirth was a tough situation, and seeing their wives in this situation would help them gain a deeper understanding of their experience: You see your baby come out a centimetre after the other, and you become very happy” (Ny et al., 2008, p. 285) *  “I must know that there are laws. I have to keep a good relation and confess that it’s I who is coming to this society” (Ny et al., 2008, p. 285)  “Not being the sole provider for the family was also hard for the men in their relationships with their wives and children, because it was difﬁcult to be a good role model if you were unable to show your children that you could take care of them ﬁnancially” (Ny et al., 2008, p. 286) *  “Here you are an extra weight to your wife. If you have money and work, you are going to teach your son to work also. You become a role model to your son” (Ny et al., 2008, p. 286) *  “The men saw themselves as the breadwinners, as having responsibility for their families” (Ny et al., 2008, p. 288) *  “The men also discussed how difﬁcult it was to be a role model for your children when you had no job and no status” (Ny et al., 2008, p. 288) *  “The different position offered to immigrant women by Swedish society affected the men’s opinion about their wives and their place in society. The wife could in some cases be perceived as a threat to the family structure and to them as men” (Ny et al., 2008, p. 288) *  “It was a major step to take for the Cameroonian men to accompany their women into the maternity unit, an unfamiliar area” (Nges et al., 2022, p. 3) *  “They found themselves emotionally unprepared when entering “the secret world” of the women, since this is an experience that does not involve male relatives in their home country” (Nges et al., 2022, p. 4)  “The fathers stated that they had to suppress their own feelings to be able to protect and support the woman. The fathers also described the hardship in putting someone else ﬁrst when you are in a state of shock and even scared of death” (Nges et al., 2022, p. 4)  “That is a special feeling, (to cut the umbilical cord) you know that haha. Its a special feeling for all men. Just ask all the men who have done it. It is very special for you to be there, I mean do that. I felt like a man, yes, I felt manly. Thats when I became a full man as I can say it even. Because I was a half man, so I became full haha. Its a big deal, yeah. Its good” (Nges et al., 2022, p. 5) *  “Back home, birthing baby is a woman’s work. And, the (birthing) woman is surrounded by other women. Here, it was my responsibility to help. I felt really awkward” (Wojnar, 2015, p. 366) *  “Somali men are not interested in the topic, that culturally appropriate education resources for both genders would attract Somali men to become fully informed and active participants in their wives’ care” (Wojnar, 2015, p. 367) *  “For both groups, the highest correlation was between physical care and care-giving, and the lowest association was the association of warmth to nurturing activities and cognitively stimulating activities” (Capps et al., 2010, p. 76)  “For Chinese immigrant fathers, social contact with friends increased the likelihood of paternal warmth with infants. These findings provide support for the notion that peer and social groups elevate the involvement of immigrant fathers, in this case Chinese fathers, and may serve as alternative role models, thereby reinforcing the importance of majority culture images and roles of the father in the household” (Capps et al., 2010, p. 84) *  “The presence of additional adults in the household was negatively associated with caregiving for Chinese immigrant fathers” (Capps et al., 2010, p. 84) *  “If it was in Afghanistan there would be no role for me as my mother would take care of it all - I wouldn’t even know about it” (Riggs et al., 2016, p. 88)  “Men reported that in Afghanistan it would be considered dishonorable or shameful for a man to attend his wife’s delivery, but in Australia men’s involvement was necessary and expected” (Riggs et al., 2016, p. 88) *  “Men reported that in Afghanistan males did not traditionally attend any aspect of maternity care, especially the birth” (Riggs et al., 2016, p. 88) *  “Generally men reﬂected that they were pleased they had the opportunity to be with their wives” (Riggs et al., 2016, p. 88)  “Being involved in pregnancy care challenged traditional values and beliefs” (Riggs et al., 2016, p. 88)  “I was happy. I wanted to help her, because being a father has responsibilities to take care of them, my culture tells me to take care of my children and family” (Riggs et al., 2016, p. 89) *  “For men in this study being involved in pregnancy, childbirth, and infant care was difﬁcult, and for” (Riggs et al., 2016, p. 90) *  “Behavioral responsibility was significantly correlated with engagement and positive machismo” (Roubinov et al., 2016, p. 283)  “Levels of paternal engagement in the current study were highest for active behaviors (taking the baby out and playing with the baby) and lowest for conventional caregiving activities (bathing, diapering). Fathers’ traditional gender role attitudes may also contribute to lower levels of paternal involvement in tasks commonly considered to be “feminine” or completed by mothers” (Roubinov et al., 2016, p. 284)  “Father-reported behavioral responsibility was correlated with positive machismo” (Roubinov et al., 2016, p. 284) |
| **Lack of extended family and building new support networks** |  | “Being physically away from their established support system in Korea, especially the wife’s mother, was acknowledged as a main challenge that the couples had to face during the out-of-culture childbirth period” (Lee, 2012, p. 118)  “We don’t have anyone to talk to when we have a hard time here. ... Hmm, and all of our close friends are in Korea. So, we don’t have anyone or any other way to talk about our difficulties and get some help from. And, I feel so nervous when I leave my wife alone at home. I am so worried when I think about, ’what if my wife faces some emergency situation when I am not there with her” (Lee, 2012, p. 119) *  “The couples mentioned that it was hard to have consistent support from their family or relatives in the U.S. because those people usually ran their own business or worked full-time” (Lee, 2012, p. 120)  “When we had our first child, I had my sister living here [in Washington]. But she could not help my wife [who did not have any helper] during the Sanhujori period because she was having a hard time raising her own two kids herself. I felt uncomfortable asking her to help us because I knew her situation” (Lee, 2012, p. 121)  "We don’t have any family here. So, I usually help my wife a lot like other American men here. ... I feel so tired once I am back from work. I know I have to, but I feel stressful sometimes" (Lee, 2012, p. 125)  “The participating couples developed close connections with at least one of three main types of local Korean community groups and their members. The types were (a) local Korean churches, (b) other local Korean immigrant families in similar age groups, and (c) newly developed web-based social support networks (e.g., kseattle.com, missyusa.com)” (Lee, 2012, p. 132)  “There was a Korean couple who experienced everything exactly one year before we did. They are one year older than us and they got married 11 months before us. They even had a baby almost one year before we did. So, we became very close. I talked to him and my wife talked to his wife whenever we have something hard to deal with. They also gave us very nice advice. We share a lot of information too. I think it is great to know those people who are in a similar situation like us, having no family here and having a hard life as immigrants. So, when we get together, we pray together and depend on each other. They were so helpful in many ways. Without them, it would have been so difficult for both of us to go through hard times” (Lee, 2012, p. 133)  “For the couples who had no help from their family members, the hardships seemed worse due to the intense workload and the limited social support available to them” (Lee, 2012, p. 193) *  “Both Congolese and Zimbabwean men said that they could not pay expensive bus or airfares for a family member to visit from Congo or Zimbabwe postpartum. Moreover, they spoke of being unable to host a female relative given their cramped living conditions, in which a family occupied just one room” (Hunter-Adams, 2016, p. 116)  “Most participants did not travel home regularly and described phone calls as brief and to-the-point because of the cost involved” (Hunter-Adams, 2016, p. 116)  “Back home after the baby is born, then the parents are there, sisters and brothers are there, then you get the whole family coming to help, you know, come and clean and do this and do that, wash the baby and everything” (Hunter-Adams, 2016, p. 116)  “You see, even if, like, she has a church where she’s a part of a church, you’ll see they will give even the announcements to the church—“Look, we’ve got our sister, she has a baby now, we have to go and visiting her, so we can’t go hands empty, so we have to bring something to her,” so they will just collect something for her, so they will bring something for her and you’ll see all, even the whole month I can say, people got enough time because they’re doing nothing, they can come, someone can come stay there from morning till late, even sleep there, spend even 2 days or 3 days, even a week, because they’ve got enough time. So here we’ve got a problem of time, so we spend too much time at work and most of our families are there, we left them far away in Congo” (Hunter-Adams, 2016, p. 116) *  “R3: Ja, I didn’t, I don’t have anyone here like to assist and take her to hospital and I had a tough time at work as well because sometimes I have to excuse myself from work and take her to hospital” (Hunter-Adams, 2016, p. 117) *  “While men presented themselves as their spouse’s primary source of support, the extent of this support had very well-deﬁned boundaries. Despite these limitations, male partners were described as a primary source of pre- and postpartum support” (Hunter-Adams, 2016, p. 118) *  “The absence of extended family in Australia left them feeling isolated and lacking in support” (Forbes et al., 2021, p. 5)  “But here ...we don’t have any extra extended family. So you are responsible for everything, for everything” (Forbes et al., 2021, p. 5)  “There would be so many people helping. Yeah. So many people to give us support. So [here] I have to be next to her, to give her support” (Forbes et al., 2021, p. 5) *  “It was common that both men and women formed strong networks of friends among the Ethiopian community” (Forbes et al., 2021, p. 5)    “Even though we are friends, but we read like we are family members.… We are very close to each other. Yeah. We support each other like at times like these” (Forbes et al., 2021, p. 6)  “Participants also re-created an extended family network through close friends they described as family” (Forbes et al., 2021, p. 10)  “Since the very first appointment with the doctor, I had always been present, which is not customary where I come from . . . Back home, as a man, if your wife is pregnant or your wife delivers nothing changes, you just continue with your life. It’s like you get a phone call, your wife has delivered, you are so happy, you tell your friends your wife is at the hospital, she has just given birth . . . nothing changes. Because usually, her mother is there with her and the husband has no role to play there” (Onyeze-Joe et al., 2022, p. 5) *  “The fathers repeatedly spoke about the absence of family support within the European context” (Onyeze-Joe et al., 2022, p. 6)  “I saw a lot of men and women taking care of their children alone and planning for everything. I knew it wouldn’t be my mum who would be assisting us in everything. It’s just going to be me, my wife, and the baby” (Onyeze-Joe et al., 2022, p. 6)  “The African fathers in our study agreed that a father’s most important role is to provide in African cultures. Hence, it is expected that the “other support” is filled by extended female family members, especially at the critical moment of birth and early child care” (Onyeze-Joe et al., 2022, p. 8) *  “In their native countries, it was the female network, with the mother-in- law in charge, that helped and took care of women during pregnancy and childbirth: In Sweden the man has taken her place” (Ny et al., 2008, p. 285) *  “Becoming integrated can be difﬁcult because of structural social problems, such as isolation within segregated areas, crowded housing and no opportunites for employment” (Ny et al., 2008, p. 286) *  “Back home, birthing baby is a woman’s work. And, the (birthing) woman is surrounded by other women. Here, it was my responsibility to help. I felt really awkward” (Wojnar, 2015, p. 366) *  “For Chinese immigrant fathers, social contact with friends increased the likelihood of paternal warmth with infants. These findings provide support for the notion that peer and social groups elevate the involvement of immigrant fathers, in this case Chinese fathers, and may serve as alternative role models, thereby reinforcing the importance of majority culture images and roles of the father in the household” (Capps et al., 2010, p. 84) *  “The presence of additional adults in the household was negatively associated with caregiving for Chinese immigrant fathers” (Capps et al., 2010, p. 85) *  “Actually, here all the time I was with my wife but in Afghanistan, my family, my father, mother and other relatives would take care of my wife and child, but here I play a hundred roles during pregnancy and appointments. I accompany her, but in Afghanistan the culture is different. Sometimes it is difﬁcult” (Riggs et al., 2016, p. 88) *  “If it was in Afghanistan there would be no role for me as my mother would take care of it all—I wouldn’t even know about it” (Riggs et al., 2016, p. 88) *  “A considerable transition for Afghan men and given the lack of extended family close-by, they were required to fulﬁll multiple roles” (Riggs et al., 2016, p. 88) *  “Men of refugee background are the key support for their wives, who have often left behind female friends, family, and relatives who would have traditionally fulﬁlled roles during pregnancy and childbirth” (Riggs et al., 2016, p. 90) * |
| **Being a main supportive figure** | **Financial responsibility** | “That is, I could not help her or spend time with her much when she had our first one because at least one of us should make money... but, I always felt so sorry for that. There was nothing I could at that time, but still, whenever I thought about that time of period, I still feel heartbroken. I always think, I should be nice to her ... really nice to her. I always think about it. By the way, we pretty much depend on each other in a large part [of our lives] ... Anyway, I am so thankful to her, a lot. [pause] She has been depending on me, trusting in me, [and] trying her best as a mother ... [brief pause] so thankful to her” (Lee, 2012, p. 131)  “The majority of mothers and fathers in this sample had less than a high school education, most of the fathers were employed but not the mothers, and half of the families reported a low annual income” (Khalil, 2022b, p. 6)  "Oh, gosh. We were confused. You know, we have to pay quite a bit [the insurance premium] every month. But, how come we have to pay, what was it? Um [pause], oh! The deductible! Gosh, it’s so confusing" (Lee, 2012, p. 142)  “Oh, especially, the ultrasound tests. During our first and second pregnancy, we could have only one ultrasound test because our insurance covered it only one time. We knew in Korea, you can have an ultrasound test whenever you go to doctor’s office if you want. But here, one time, and that’s it. So, I became so nervous especially at the end of pregnancy because I wanted to see my baby with my eyes and make sure (s)he was growing well inside. But when I asked [health care providers], they said I had to pay everything out of my pocket for that. And it was, wow, too expensive for us. It is so different from Korea and we really did not like it at all” (Lee, 2012, p. 148)  “The traditional role of Korean husbands is that of family financial supporter” (Lee, 2012, p. 192)  “For the couples who had no help from their family members, the hardships seemed worse due to the intense workload and the limited social support available to them” (Lee, 2012, p. 193)  “So here we’ve got a problem of time, so we spend too much time at work and most of our families are there, we left them far away in Congo” (Hunter-Adams, 2016, p. 1016)  “Rather, participants emphasized the pressure to survive ﬁnancially and to provide for family members back home; they generally self-categorized as very busy and unable to offer real support to one another” (Hunter-Adams, 2016, p. 1017)  “When they could ﬁnd a car to use and ﬁnancial provider, all while having to take an active role in raising their new baby” (Hunter-Adams, 2016, p. 1018)  “Men in all three focus groups described the ﬁnancial and physical burden of having a wife or girlfriend who was pregnant” (Hunter-Adams, 2016, p. 1021) *  “Pressure, obligation, and support were intertwined, and while men and women articulated loss, it was also clear that they were physically and ﬁnancially unable to provide extensive supports to others” (Hunter-Adams, 2016, p. 1021) *    “The reciprocal and extensive set of physical, cognitive, and emotional supports had real-time costs, making it difﬁcult for migrants to reestablish these links in a new country, particularly given the continued ﬁnancial commitments to family at home” (Hunter-Adams, 2016, p. 1021) *  “Several men said they would have liked to attend more perinatal health appointments but were not able to because of work commitments” (Forbes et al., 2021, p. 8)  “Men described the responsibility of being the breadwinner and also attempting to take care of their partners and children physically and emotionally through the perinatal period, as challenging and stressful” (Forbes et al., 2021, p. 8) *  “The key barrier in this study to engaging in perinatal healthcare among men was paid employment, lack of flexible working conditions and insufficient paternity leave” (Forbes et al., 2021, p. 10)  “The fathers acknowledged being coplayers with their partners at each stage from pregnancy, birth, and in child care. Even though they strongly believed in their role as breadwinners, they were willing to also embrace domestic and child care responsibilities as well” (Onyeze-Joe et al., 2022, p. 7) *  “To spend time with your children, being honest and having a job were important aspects of the father’s role. The father’s personality, and society’s perception of the role of the man, affects how a person acts as a father, both in their native country and also in Sweden” (Ny et al., 2008, p. 285)  “Their relationships with their wives and children, because it was difﬁcult to be a good role model if you were unable to show your children that you could take care of them ﬁnancially” (Ny et al., 2008, p. 286) *  “Here you are an extra weight to your wife. If you have money and work, you are going to teach your son to work also. You become a role model to your son” (Ny et al., 2008, p. 286) *    “The men saw themselves as the breadwinners, as having responsibility for their families” (Ny et al., 2008, p. 288) *  “Our results showed that higher levels of acculturative stress reported by fathers related to higher levels of PPD symptoms reported by mothers. One of the main roles of the father in the Arab American culture is to provide ﬁnancial support and the sense of security in the family” (Khalil et al., 2022a, p. 96)  “Those cultural expectations along with the growing responsibilities during immigration and postpartum period may increase the emphasis on the father’s role as the ﬁnancial provider and may create psychological distress contributing to the development of stress and depression among couples” (Khalil et al., 2022a, p. 96)    “Having ﬁnancial security to support their wives, newborn baby, and other children was important to all of the men. Yet employment also proved to be a dilemma for men who were anxious about their availability to support their wives” (Riggs et al., 2016, p. 88)  “Higher levels of engagement were reported by fathers when mothers worked partor full-time as compared with men with partners who did not work outside the home, r ⫽ .39, p ⬍ .01” (Roubinov et al., 2016, p. 283)  “Fathers self-reported higher engagement when their partners were employed part- or full-time. It may be the case that employed mothers have less time to participate in direct interaction and caregiving activities, calling forth increased engagement from fathers” (Roubinov et al., 2016, p. 285) |
|  | **Main supportive role during pregnancy, childbirth, and postpartum** | “Over time, fathers’ understanding of and interactions with their partners seemed to adjust as they became more cognizant of the stressors in family life that contributed to the mothers’ mental state” (Valdez & Martinez, 2019, p. 312)  “Fathers talked about “sneaking out of work” at lunchtime to help partners with errands or simply to lend emotional support” (Valdez & Martinez, 2019, p. 313)  “Fathers seemed to increase involvement in the home not only to support the mother but also to enhance marital exchanges” (Valdez & Martinez, 2019, p. 313) *  “The fathers conveyed that their previous expectations for the mothers to fulﬁll most household and caregiving roles were harmful to their partners’ wellbeing and rooted in traditional gender norms of machismo” (Valdez & Martinez, 2019, p. 314)  “They indicated coming out of the experience of their partner’s depression feeling empowered by their increased role within the family” (Valdez & Martinez, 2019, p. 315)  “Fathers said they worried about the safety of their partners and that this worry made them weary of leaving the house in the mornings” (Valdez & Martinez, 2019, p. 316) *  “Fathers in our case studies indicated that they changed their behavior and roles in tandem with their better understanding of maternal depression. They concurred that receiving accurate information about maternal depression was key to understanding it, which, in turn, allowed them to manage their emotional responses and to overcome constraints of traditional machismo to support the family” (Valdez & Martinez, 2019, p. 317) *  “Traditionally, husbands were not allowed to be present during their wives’ labor in Korea. Findings in this study suggest that all of the participating husbands felt that they were expected to take an active role during the labor in the U.S. health culture” (Lee, 2012, p. 124)  "We don’t have any family here. So, I usually help my wife a lot like other American men here. ... I feel so tired once I am back from work. I know I have to, but I feel stressful sometimes" (Lee, 2012, p. 125) *  “Sometimes, I feel it is hard [to do housework]. We cannot afford to hire a housekeeper. I have to do it [housework], otherwise my wife has to do all the work” (Lee, 2012, p. 126) *  “Compared to her [wife] hard work at home ... but still, I was busy at work too but I had to help her with cleaning or laundry stuff. And, gosh, we had to wake up at night and could not sleep much. ... We had really difficult time for a while” (Lee, 2012, p. 126) *  “I really wanted to do my best to get whatever she wanted to eat during her pregnancy. I thought, at least, it was all I could do for her. But one day, she said, in the middle of night, she wanted to eat a kind of Korean shell-fish soup. You know, there was no way for me to buy it here. Then, she could not even sleep well for a couple of days because she wanted to eat that soup so badly. I heard that when a pregnant woman cannot eat what she really wants to eat, she can even get sick from it. Oh, Gosh. I was very frustrated because there was nothing I could do about it” (Lee, 2012, p. 127) *  “All husbands considered their experience as an active partner or a labor coach to be a life-changing one” (Lee, 2012, p. 130) *  “Rather, participants emphasized the pressure to survive ﬁnancially and to provide for family members back home; they generally self-categorized as very busy and unable to offer real support to one another” (Hunter-Adams, 2016, p. 1017) *  “Partners, who felt that they were responsible for more than they could handle, self-identiﬁed as their partner’s driver” (Hunter-Adams, 2016, p. 1017) *  “It’s quite a big deal when it comes to being a husband.…It’s quite scary.…Your wife is pregnant now, then from that moment on it becomes, it remains your responsibility, according to our culture, in terms of helping the wife” (Hunter-Adams, 2016, p. 1018) *  “All the time and any time she needs help and taking her to hospital and all this stuff, assisting her because she didn’t know English as well and she has to go, before she goes to hospital I have to write everything in a paper and when you go you have to say, “Give them the paper, he will read the paper.” So when they do all the tests, then I ask her also to ask the nurse to write in the paper so I can understand and explain to her at home” (Hunter-Adams, 2016, p. 1018)  “Ja, I didn’t, I don’t have anyone here like to assist and take her to hospital and I had a tough time at work as well because sometimes I have to excuse myself from work and take her to hospital” (Hunter-Adams, 2016, p. 1018) *  “Men from all three migrant groups described themselves as solely responsible for caring for their pregnant wives” (Hunter-Adams, 2016, p. 1018)  “Some men did discuss the health of the baby separately from the health of their partners, and they felt a duty to both their partner and their unborn child” (Hunter-Adams, 2016, p. 1018) *  “You know, a pregnant woman, she’s like a small child, a baby, that’s what is a pregnant woman, that’s the example that I can give to a pregnant woman: a child. If you’ve got a child, you are the one who choose the food for her. Sometimes she don’t want that food and you don’t know what she want, so same applies to your woman” (Hunter-Adams, 2016, p. 1019) *  “While men presented themselves as their spouse’s primary source of support, the extent of this support had very well-deﬁned boundaries. Despite these limitations, male partners were described as a primary source of pre- and postpartum support” (Hunter-Adams, 2016, p. 1019) *  “There would be so many people helping. Yeah. So many people to give us support. So [here] I have to be next to her, to give her support” (Forbes et al., 2021, p. 5) *  “They wanted to offer emotional support to their partner, and they felt going together supported the relationship” (Forbes et al., 2021, p. 7)  “During pregnancy something happens, I have to support her. Yeah. I was very happy to support her all the time” (Forbes et al., 2021, p. 7)  “They also performed a functional role through driving to the appointments, navigating Australian health services and helping to interpret during healthcare consultations” (Forbes et al., 2021, p. 7)  “It was more likely men would take time off to attend antenatal check-ups if their partner was unwell or there were concerns or complications with the pregnancy” (Forbes et al., 2021, p. 8) *  “Men described the responsibility of being the breadwinner and also attempting to take care of their partners and children physically and emotionally through the perinatal period, as challenging and stressful” (Forbes et al., 2021, p. 8) *  “Participants said there was a lot of work involved in pregnancy, childbirth and raising children and without support, this would be too much for one person” (Forbes et al., 2021, p. 8)  “You are responsible for everything from pregnancy and childbirth and then raising children. So it would be very, very, very difficult for a single person” (Forbes et al., 2021, p. 8) *  “Participants also said it was important for men to be involved in the perinatal period overall, to support fathers’ bonding and relationship with their children” (Forbes et al., 2021, p. 9) *  “If you participate in everything, then I think you feel that you oh, you put in everything. It’s a lot better. So it’s for you, for your partner, for the baby. It would be very good to involve fathers in every aspect” (Forbes et al., 2021, p. 9)  “Men performed roles during perinatal healthcare that included emotional support, logistic support (language translation or driving) and being a part of the shared decision making process” (Forbes et al., 2021, p. 9)  “All participants believed that their major role is to support their partners. “The main thing is being supportive because she can’t do it all alone” (Onyeze-Joe et al., 2022, p. 5) *  “I wasn’t doing my regular fun activities like watching football, doing the things I loved to do. All I did was what I could do at that moment. I was there for my wife and the baby. Before then, I went to play football like twice a week, I could not do that anymore. All my fun activities just ended with my new reality” (Onyeze-Joe et al., 2022, p. 5) *  “The fathers acknowledged being coplayers with their partners at each stage from pregnancy, birth, and in child care. Even though they strongly believed in their role as breadwinners, they were willing to also embrace domestic and child care responsibilities as well” (Onyeze-Joe et al., 2022, p. 7) *  “The African fathers in our study agreed that a father’s most important role is to provide in African cultures. Hence, it is expected that the “other support” is filled by extended female family members, especially at the critical moment of birth and early child care” (Onyeze-Joe et al., 2022, p. 8) *  “I see in this society that I have to go with my daughter son or wife, side by side, I must know” (Ny et al., 2008, p. 285)  “Fathers found themselves bonding naturally with their women and babies. Most importantly, they felt more connected to their families and nothing else mattered at that point, just their babies and their women” (Nges et al., 2022, p. 5) *  “Somali men are not interested in the topic, that culturally appropriate education resources for both genders would attract Somali men to become fully informed and active participants in their wives’ care” (Wojnar, 2015, p. 367) *  “Paternal acculturative stress had a signiﬁcant moderate positive correlation with mothers’ PPD symptoms (r = .39, p = .04)” (Khalil et al., 2022a, p. 96) *  “Actually here all the time I was with my wife but in Afghanistan, my family, my father, mother and other relatives would take care of my wife and child, but here I play a hundred roles during pregnancy and appointments. I accompany her, but in Afghanistan the culture is different. Sometimes it is difﬁcult” (Riggs et al., 2016, p. 88) *  “A considerable transition for Afghan men and given the lack of extended family close-by, they were required to fulﬁll multiple roles” (Riggs et al., 2016, p. 88) *  “Generally, men reﬂected that they were pleased they had the opportunity to be with their wives” (Riggs et al., 2016, p. 88)  “I was happy. I wanted to help her, because being a father has responsibilities to take care of them, my culture tells me to take care of my children and family” (Riggs et al., 2016, p. 89)  “Men talked of the expectations placed on them to assist their wives to attend appointments and have the necessary pregnancy tests” (Riggs et al., 2016, p. 89)    “I was alone with her and she was new in Australia, she was not aware.. .where to ﬁnd the GP and the need to do an ultrasound. I was the only one able to assist” (Riggs et al., 2016, p. 89)  “I had a big role in this. I kept her company and was always there with her to help her and support her” (Riggs et al., 2016, p. 89)  “As a father it’s my duty to help my wife and be with her .. . she couldn’t drive or speak English so I went with her to help her” (Riggs et al., 2016, p. 89)  “I would be less responsible there [in Afghanistan] as my wife would be able to speak the language and go see the doctor as here I have to go with her and drive as well. Even during labor” (Riggs et al., 2016, p. 89)  “Men of refugee background are the key support for their wives, who have often left behind female friends, family, and relatives who would have traditionally fulﬁlled roles during pregnancy and childbirth” (Riggs et al., 2016, p. 90) *  “Fathers self-reported higher engagement when their partners were employed part- or full-time. It may be the case that employed mothers have less time to participate in direct interaction and caregiving activities, calling forth increased engagement from fathers” (Roubinov et al., 2016, p. 285) *  “It is different, you know, different from just saying, ’Oh, you did it. Great job, Honey,’ without seeing anything about labor. It could be so different. And when you see everything right there, I mean, you felt her pain during the labor and saw how hard it was. I was so emotional at that time [in emotional tone] ... I still remember everything even now. It came to my mind that I should be good to my wife. If you go through labor together, you cannot be bad to your wife. No, you just can’t, if you saw everything there” (Lee, 2012, p. 130)  “Some men did discuss the health of the baby separately from the health of their partners, and they felt a duty to both their partner and their unborn child” (Hunter-Adams, 2016, p. 1018) *  “It was quite common for male partners to attend antenatal care in Australia. The reasons given were frequently related to emotional support, logistical support like translating English language or driving and to support their wife because she felt unwell” (Forbes et al., 2021, p. 6)  “Men described the responsibility of being the breadwinner and also attempting to take care of their partners and children physically and emotionally through the perinatal period, as challenging and stressful” (Forbes et al., 2021, p. 8) *  “I think it is good for every father to see that and how a woman’s suffering during birth. It’s very nice to watch and how they give birth. It’s very, very tough moment for them...To bring good family, to grow up with a good family it needs bond. A good bond with a wife to look after each other” (Forbes et al., 2021, p. 8)  “Men should be involved to help them understand the suffering and challenge involved in pregnancy, birth and caring for children” (Forbes et al., 2021, p. 8) *  “Participants also said it was important for men to be involved in the perinatal period overall, to support fathers’ bonding and relationship with their children” (Forbes et al., 2021, p. 9) *  “Several men mentioned that participating in pregnancy and birth opened up a new world for them” (Ny et al., 2008, p. 285)  “They attended midwife and child health-care nurse appointments with their wives, and often acted as interpreter” (Ny et al., 2008, p. 285) *  “It was a major step to take for the Cameroonian men to accompany their women into the maternity unit, an unfamiliar area” (Nges et al., 2022, p. 4) *  “The Cameroonian fathers in Sweden felt pressured to be there (labour and childbirth), but at the same time, being the only relative in a new country one had no choice but to participate” (Nges et al., 2022, p.4)  “I was a little bit nervous because I knew that was not my position, I knew that it wasn’t my place, from a cultural standpoint. But having spent time here, having been educated here and also having had that experience here I knew that I had to be able to… come along” (Nges et al., 2022, p.4) *  “Fathers stated that being there as the closest person to their women and seeing her undergo severe and enduring pain was traumatizing and brought feelings of frustration. However, being there to comfort and share the hardship with the woman gave a sense of meaningfulness to the fathers” (Nges et al., 2022, p.4) *  “It was a good… good, bad situation. But towards the end, the baby is here, and she is healthy…(...)...that’s most important” (Nges et al., 2022, p.4) *  “I can’t be tired! ...I was a little tired, but not as tired as her. And at the same time, I felt that ... it is my responsibility to make sure she will calm down. Because if I show that I am tired or that I am afraid of what will happen, then she becomes even more afraid” (Nges et al., 2022, p.4) *  “Fathers found themselves bonding naturally with their women and babies. Most importantly, they felt more connected to their families and nothing else mattered at that point, just their babies and their women” (Nges et al., 2022, p.5) *  “In spite of the emotional diﬃculties encountered during labour and childbirth, the fathers were not discouraged. Having found their role in the womans’ world, obtaining new knowledge and a better understanding of the values of life, they still had a positive apprehension of being a part of the process” (Nges et al., 2022, p.5) *  “The results concerning having anxiety until the baby was born; the need for information about childbirth; ﬁnding their supportive role; as well as experiencing an emotional and learning moment that was joyful” (Nges et al., 2022, p.5) *  “When our last son was born, I went to hospital with her because there were no women to go with her. I knew some English but my she didn’t and we had a computer translation. But, when the baby came out, they turned it off. They took my baby away and gave him shots. I thought something was wrong with him and I started crying. When the nurse saw it she asked: “Are you crying because you are happy?” and I said “No, what is wrong with my baby?” and she said “Nothing, he is getting normal care.” I wanted to hold the baby ﬁrst and say a blessing like I did back home but I didn’t know how to ask or if I could. I wish I had the opportunity to do it my way. I just want them to accept and respect us just as we are, but I didn’t know how to say it....” (Wojnar, 2015, p. 363 - 364) *  “Back home, birthing baby is a woman’s work. And, the (birthing) woman is surrounded by other women. Here, it was my responsibility to help. I felt really awkward” (Wojnar, 2015, p. 363) *  “Male participants wanted to talk about it during their individual interviews to explore new ways of alleviating their wives’ pain and discomfort or admitted their wives had reconstructive surgery of the perineum” (Wojnar, 2015, p. 367) *  “Men reported that in Afghanistan it would be considered dishonorable or shameful for a man to attend his wife’s delivery, but in Australia men’s involvement was necessary and expected” (Riggs et al., 2016, p. 88) *    “The fact that I was there with my wife during labor [was good], because I had never seen someone giving birth so I could understand her pain and also my wife was happy that I was there standing by her” (Riggs et al., 2016, p. 89)  “An overarching concern for men was having access to female care providers and being interpreters for their wives” (Riggs et al., 2016, p. 89)  “Language support in labor was usually provided by husbands, although their level of English was often insufﬁcient” (Riggs et al., 2016, p. 89) *  “I would be less responsible there [in Afghanistan] as my wife would be able to speak the language and go see the doctor as here I have to go with her and drive as well. Even during labor” (Riggs et al., 2016, p. 89) * |
| * extract coded to more than one theme | | |

**First Order Theme: Fathers’ needs and personal difficulties**

| **Second order theme** | **Third order theme** | **Data codes** |
| --- | --- | --- |
| **Information** |  | “Some fathers described their partners’ states as extensions of normal feelings and behaviors, such as crying and sleeping, but struggled to make sense of their intensity and pervasiveness” (Valdez & Martinez, 2019, p. 311)  “Regardless of whether fathers expressed concern over their partners during the ﬁrst interviews, the majority of fathers did not initially associate the behavior with depression” (Valdez & Martinez, 2019, p. 312)  “One father expressed, “It’s really difﬁcult to understand the person who is going through it. It’s easier for you to... like get angry, then the arguments start, the whole problem starts... because there is no way that you have understood it” (Valdez & Martinez, 2019, p. 312) *  “Fathers described stigma about mental illness as blocking their initial understanding and recognized the role of accurate information about depression as shifting their awareness and approach” (Valdez & Martinez, 2019, p. 314)  “I think the prenatal classes were so helpful to me. We took many different classes. There was a class about newborn baby care and another one about preparing for labor. And I could get used to some medical words in English while I took that class because they informed us about those [medical] words in the class. So, it was easier [for us] to understand later when we went to hospital for labor. While I was taking those classes, I had a chance to change diapers by myself and watched a video about the labor process for the first time in my life. ... It was kind of shocking. I think, it could have been more shocking me if we went into the labor without watching it [before]. And, there was a class for the hospital tour in advance. I liked it a lot too” (Lee, 2012, p. 139)  "Oh, gosh. We were confused. You know, we have to pay quite a bit [the insurance premium] every month. But, how come we have to pay, what was it? Um [pause], oh! The deductible! Gosh, it’s so confusing" (Lee, 2012, p. 142) *  "Our OB doctor didn’t tell us much about newborn baby there [the circumcision site]. So, [we] asked around or searched on the websites. But, still, not easy with those medical words. [I] just wanted to be prepared for the emergency with my baby" (Lee, 2012, p. 143)  “It was most difficult for us to not know much about the labor progress. We did not have any idea about the progress because it was our first labor experience. So, I became very passive. When the nurse came in and checked on her I thought, ’oh, it is time to check her’. When she gave her a shot, then I thought, ’oh, she needed to get a shot’. ... But I felt like ... everything was controlled by someone else. I don’t think that they explained well about her progress to us. We did not have much information before we got into labor. Partly due to the language barrier and partly due to the lack of information [about labor process] in advance, when we were actually in labor, we had to go through a very hard time” (Lee, 2012, p. 142)  “They felt somewhat ignored when their U.S. health care providers were not willing to give them advice on things for which they asked their opinion” (Lee, 2012, p. 146) *  “The Korean immigrant husbands of this study seemed to be struggling to help their wives in labor and during the postpartum period. Having limited support systems in the U.S. also contributed to this stressful struggle” (Lee, 2012, p. 191) *  “Participants suggested that including men supported the couple’s relationship and the father’s bond with their child, allowed men to understand the processes involved with pregnancy, childbirth and infant-care and improve their health literacy” (Forbes et al., 2021, p. 11)  “Many fathers acknowledged how unprepared they were for what they experienced” (Onyeze-Joe et al., 2022, p. 4)  “They acknowledged their awkwardness when shopping for the unborn or at information sessions where they practiced baby feeding or diaper changing a dummy baby” (Onyeze-Joe et al., 2022, p. 6)  “Several of the men stated that ‘too much’ information and information about risks could lead to anxiety” (Ny et al., 2008, p. 284)  “The thing that was strange for us is that the child should take AD-drops for about 6 years!! We went back and asked her, do you really mean 6 years? And she said yes, 6 years” (Ny et al., 2008, p. 284)  “Negative feelings took over due to the lack of knowledge about the stages of labour as well as maternity wards routines” (Nges et al., 2022, p. 4)  “The fathers felt that their interaction with their women and their contribution during the birth process was sometimes not enough to meet the expectations of the midwives” (Nges et al., 2022, p. 4)  “This was done mostly through individual research because preparatory courses were rarely offered. That notwithstanding, the fathers stated that the level of information and knowledge obtained before the childbirth never seemed to be enough, since the labour and childbirth is an unpredictable process” (Nges et al., 2022, p. 5)  “The results concerning having anxiety until the baby was born; the need for information about childbirth; ﬁnding their supportive role; as well as experiencing an emotional and learning moment that was joyful” (Nges et al., 2022, p. 5)  “The majority of couples felt uniformed and wished more time was spent on explaining things and verifying wishes and understandings” (Wojnar, 2015, p. 363)  “They hoped the caregivers would somehow know when to provide them with information without making assumptions. Because the participants often didn’t understand what was going on, they missed opportunities that were important to them for religious and cultural reasons” (Wojnar, 2015, p. 363)  “When our last son was born, I went to hospital with her because there were no women to go with her. I knew some English but my she didn’t and we had a computer translation. But, when the baby came out, they turned it off. They took my baby away and gave him shots. I thought something was wrong with him and I started crying. When the nurse saw it she asked: “Are you crying because you are happy?” and I said. “No, what is wrong with my baby?” and she said “Nothing, he is getting normal care.” I wanted to hold the baby ﬁrst and say a blessing like I did back home but I didn’t know how to ask or if I could. I wish I had the opportunity to do it my way. I just want them to accept and respect us just as we are, but I didn’t know how to say it...” (Wojnar, 2015, p. 363) *  “Male participants tended to be less concerned about their wife getting the “right” episiotomy and more concerned about them not having the cesarean birth. They consistently indicated that the memories of “women all the time dying in surgery back home” was one of the key reasons they opposed cesarean birth. Some also expressed concerns that cesarean section would limit the number of children they wanted” (Wojnar, 2015, p. 364) *  “They talked at length about the confusion and fears caused by the lack of mutual understanding at any point. Some couples felt pressured to agree to treatments whereas others felt that few providers took time to explain the informed consent to their full satisfaction and understanding” (Wojnar, 2015, p. 364) *  “The longer you are in this country, the smarter you become about the differences between you and the Americans and about your rights. You begin to understand that you have to be your own advocate in health care because nobody else will. It is very true when you go to hospital to have a baby. If you don’t ask the right questions they will assume you understand and agree to everything while you don’t” (Wojnar, 2015, p. 365) *  “Before we went home, the nurse asked me how I was doing. I was relieved she asked because I wasn’t doing well at all. She then explained and showed me how to do different things. It helped me realize how much help my wife will need after we get home because she had a c-section” (Wojnar, 2015, p. 366)  “For men, trust was an important and positive element in their relationships and interactions with care providers. Everything is good in pregnancy care because we trust them” (Riggs et al., 2016, p. 89) *  “The majority of men were not asked by health professionals about their circumstances including ﬁnancial worries, legal and housing problems” (Riggs et al., 2016, p. 90) *  “There was little recognition that men may require information or advice or have questions. A few men were able to rely on social networks to resolve issues concerning them” (Riggs et al., 2016, p. 90)  “We started our life from zero, and wanted information on how to start work” (Riggs et al., 2016, p. 90)  “Men were seeking support from services, as they were unsure where else they could ﬁnd assistance” (Riggs et al., 2016, p. 91) |
| **Emotional and psychological challenges** |  | “Additionally, a high percentage of mothers and fathers in our sample met diagnostic criteria for depression, 24% and 35%, respectively” (Khalil et al., 2023, p. 142)  “Fathers expressed concern during the ﬁrst interviews about what they observed to be the mothers’ deteriorating emotional and behavioral state” (Valdez & Martinez, 2019, p. 311)  “Some fathers described their partners’ states as extensions of normal feelings and behaviors, such as crying and sleeping, but struggled to make sense of their intensity and pervasiveness” (Valdez & Martinez, 2019, p. 312)  “The men expressed frustration when they perceived the women as not making enough effort to improve” (Valdez & Martinez, 2019, p. 312)  “Although these varied strategies seemed to alleviate mothers’ symptoms, they did not fully help them manage depression, which led to fathers’ feeling helpless or frustrated” (Valdez & Martinez, 2019, p. 312)*  “One father expressed, “It’s really difﬁcult to understand the person who is going through it. It’s easier for you to... like get angry, then the arguments start, the whole problem starts... because there is no way that you have understood it.” (Valdez & Martinez, 2019, p. 312) *  “Many (the fathers) recounted lingering feelings of anxiety and helplessness, loneliness and shame, and fear and concern” (Valdez & Martinez, 2019, p. 315)  “Fathers said they countered the helplessness they felt with their partners’ depression by focusing on their children, which gave them a sense of mastery and enjoyment” (Valdez & Martinez, 2019, p. 315)  “Fathers said they also took care of themselves. One father talked about the importance of physical exercise in staying healthy. Another talked about outside activities and their bond to friends and extended family as a source of strength” (Valdez & Martinez, 2019, p. 315)  “Fathers paired prolonged anxiety about family climate with feelings of helplessness or powerlessness” (Valdez & Martinez, 2019, p. 315)  “One father exempliﬁed the burden of helplessness during his partner’s recovery based on the unpredictability of family life: “One feels impotent to plan things...tobe unable to go out, from one day to the next, that’s the most frustrating for me” (Valdez & Martinez, 2019, p. 315)  “These worries made fathers at times internally question their commitment: “There comes a time when you reach your limit and you can’t be part of a toxic relationship anymore. What happens? You start to dread coming home and leaving to get away from that situation” (Valdez & Martinez, 2019, p. 315)  “Fathers often described feeling lonely as they coped with their partners’ depression” (Valdez & Martinez, 2019, p. 315)  “For other fathers, loneliness resulted from not knowing whether their supportive efforts were working” (Valdez & Martinez, 2019, p. 316)  “Sometimes I just try to help her... But I feel bad... because there are times when you think that... your help has been useless... that’s when I think you start to feel desperate” (Valdez & Martinez, 2019, p. 316)  “They felt frustrated with the slow pace of recovery, a few fathers said they contemplated leaving their partners” (Valdez & Martinez, 2019, p. 316)  “Fathers said they worried about the safety of their partners and that this worry made them weary of leaving the house in the mornings” (Valdez & Martinez, 2019, p. 316)  “Many fathers lived in a state of confusion and anxiety about their partners’ emotional state, which contributed to frequent couple and family discord” (Valdez & Martinez, 2019, p. 317)  “Fathers expressed was that they experience a heavy psychological burden in caring for their partners and families, even as they report improved family life and a sense of mastery from their role in supporting the family” (Valdez & Martinez, 2019, p. 318) *  “Fathers have difﬁculty managing the emotional toll of being caregivers, often feeling constrained and cautious, and exhausted and overwhelmed when their partners have depression” (Valdez & Martinez, 2019, p. 318)  “We don’t have anyone to talk to when we have a hard time here. ... Hmm, and all of our close friends are in Korea. So, we don’t have anyone or any other way to talk about our difficulties and get some help from. And, I feel so nervous when I leave my wife alone at home. I am so worried when I think about, ’what if my wife faces some emergency situation when I am not there with her” (Lee, 2012, p. 199) *  “I am just an ordinary guy. It was not easy to see that much of blood at once. That part ... was hard. And, there was too much discharge. When our baby was born, she was covered with blood too. Even when I cut the cord, there was blood oozing out from it. That was a little bit... and uh... but at that time though, because I was too excited to think of anything else but we just had a baby. So I did not feel much anything about it [blood]. But later on, when I recalled the labor room, oh ... my, my. I think it was not easy to deal with the bloody scene” (Lee, 2012, p. 124) *  "We don’t have any family here. So, I usually help my wife a lot like other American men here. ... I feel so tired once I am back from work. I know I have to, but I feel stressful sometimes" (Lee, 2012, p. 125) *  “Sometimes, I feel it is hard [to do housework]. We cannot afford to hire a housekeeper. I have to do it [housework], otherwise my wife has to do all the work” (Lee, 2012, p. 126) *  “Compared to her [wife] hard work at home ... but still, I was busy at work too but I had to help her with cleaning or laundry stuff. And, gosh, we had to wake up at night and could not sleep much. ... We had really difficult time for a while” (Lee, 2012, p. 126) *  “I really wanted to do my best to get whatever she wanted to eat during her pregnancy. I thought, at least, it was all I could do for her. But one day, she said, in the middle of night, she wanted to eat a kind of Korean shell-fish soup. You know, there was no way for me to buy it here. Then, she could not even sleep well for a couple of days because she wanted to eat that soup so badly. I heard that when a pregnant woman cannot eat what she really wants to eat, she can even get sick from it. Oh, Gosh. I was very frustrated because there was nothing I could do about it” (Lee, 2012, p. 127) *  “Moreover, when we decided to have a family and a baby here, I felt so much pressure on my shoulders ... but I should be in charge and do my best. I am the head of household here ... Everyone depends on me. I don’t know whether it is because I am simply getting old or because I am being more mature. But I just think about the responsibility [as the head of household] a lot” (Lee, 2012, p. 129) *  “That is, I could not help her or spend time with her much when she had our first one because at least one of us should make money... but, I always felt so sorry for that. There was nothing I could at that time, but still, whenever I thought about that time of period, I still feel heartbroken. I always think, I should be nice to her ... really nice to her. I always think about it. By the way, we pretty much depend on each other in a large part [of our lives] ... Anyway, I am so thankful to her, a lot. [pause] She has been depending on me, trustting in me, [and] trying her best as a mother ... [brief pause] so thankful to her” (Lee, 2012, p. 131) *  “Her husband said, "She became so fragile. I don’t know much, but she has been suffering  from Sanhupoong. I feel sorry. I told her to go to Korea for a while until she got her health back” (Lee, 2012, p. 164)  “Six husbands of these 12 wives mentioned that they also had a hard time dealing with  their wives’ depressive symptoms. Husband 15 stated, "She was a totally different person right after [she] had the baby. She usually is a very happy person. But she looked so sad and down for a while, so I worried a lot" (Lee, 2012, p. 165)  “The Korean immigrant husbands of this study seemed to be struggling to help their wives in labor and during the postpartum period. Having limited support systems in the U.S. also contributed to this stressful struggle” (Lee, 2012, p. 191) *  “One unexpected finding is the Korean husbands’ expressed hardships during their wives’ Sanhujori period” (Lee, 2012, p. 193)  “Partners, who felt that they were responsible for more than they could handle, self-identiﬁed as their partner’s driver” (Hunter-Adams, 2016, p. 1017)  “It’s quite a big deal when it comes to being a husband.…It’s quite scary.…Your wife is pregnant now, then from that moment on it becomes, it remains your responsibility, according to our culture, in terms of helping the wife” (Hunter-Adams, 2016, p. 1018)  “Navigating their wife’s needs was often described as physically and ﬁnancially burdensome” (Hunter-Adams, 2016, p. 1018)  “Men in all three focus groups described the ﬁnancial and physical burden of having a wife or girlfriend who was pregnant” (Hunter-Adams, 2016, p. 1021) *  “Pressure, obligation, and support were intertwined, and while men and women articulated loss, it was also clear that they were physically and ﬁnancially unable to provide extensive supports to others” (Hunter-Adams, 2016, p. 1021) *  “You go inside the maternity room and you see when the baby comes out. That’s not in back home, they’d be coming out only the midwives would go with them….. [Here] they allow me to cut the umbilical cord and cut with the scissors. Yep It’s good and it’s a bit painful to see all that drama” (Forbes et al., 2021, p. 5) *  “You are responsible for everything from pregnancy and childbirth and then raising children. So it would be very, very, very difficult for a single person” (Forbes et al., 2021, p. 8) *  “I think it is good for every father to see that and how a woman’s suffering during birth. It’s very nice to watch and how they give birth. It’s very, very tough moment for them...To bring good family, to grow up with a good family it needs bond. A good bond with a wife to look after each other” (Forbes et al., 2021, p. 8) *  “In this study, a large percentage of parents reported clinically significant  symptoms of anxiety (33% mothers; 45% fathers), and depression  (33% mothers; 35.5% fathers)” (Khalil et al., 2022b, p. 5)  “Most of the fathers spoke about the control they had over their social lives, the freedom to go anyway and be anywhere and plan their time with no extra responsibilities” (Onyeze-Joe et al., 2022, p. 4)  “They spoke about the loss of personal freedom to the increasing daily commitments and responsibilities during the pregnancy and then as fathers” (Onyeze-Joe et al., 2022, p. 5) *  “I wasn’t doing my regular fun activities like watching football, doing the things I loved to do. All I did was what I could do at that moment. I was there for my wife and the baby. Before then, I went to play football like twice a week, I could not do that anymore. All my fun activities just ended with my new reality” (Onyeze-Joe et al., 2022, p. 5) *  “Although challenging at first, many of the fathers were able to adjust to their new role with time and even prioritized their family time over other personal needs” (Onyeze-Joe et al., 2022, p. 7) *  “Several of the men stated that ‘too much’ information and information about risks could lead to anxiety” (Ny et al., 2008, p. 284) *  “Most liked receiving information in groups, although found some of the bad experiences dicussed by the other men frightening” (Ny et al., 2008, p. 284)  “I don’t encourage such meetings because the women begin to frighten each other, and the woman feels psychologically tiredy. Even the man becomes afraid, just like the woman. Let’s keep it natural” (Ny et al., 2008, p. 284) *  “As a result of increased participation, the men felt respect for the women during childbirth. They could see that the women and their babies were treated kindly and were offered medical resources” (Ny et al., 2008, p. 284)  “They acknowledged that childbirth was a tough situation, and seeing their wives in this situation would help them gain a deeper understanding of their experience: You see your baby come out a centimetre after the other, and you become very happy” (Ny et al., 2008, p. 285) *  “The Cameroonian fathers in Sweden felt pressured to be there (labour and childbirth), but at the same time, being the only relative in a new country one had no choice but to participate” (Nges et al., 2022, p. 4) *  “Despite the feelings of (partly) being pressurized, the fathers’ involvement was also (partly) voluntary, and they expressed feelings of being honoured and appreciated the opportunity to be part of the birth of their child” (Nges et al., 2022, p. 4)  “They found themselves emotionally unprepared when entering “the secret world” of the women, since this is an experience that does not involve male relatives in their home country” (Nges et al., 2022, p. 4)  “I was a little bit nervous because I knew that was not my position, I knew that it wasn’t my place, from a cultural standpoint. But having spent time here, having been educated here and also having had that experience here I knew that I had to be able to… come along” (Nges et al., 2022, p. 4) *  “Fathers stated that being there as the closest person to their women and seeing her undergo severe and enduring pain was traumatizing and brought feelings of frustration. However, being there to comfort and share the hardship with the woman gave a sense of meaningfulness to the fathers” (Nges et al., 2022, p. 4) *  “Feelings were so intense that fathers could relive the situation during the interviews” (Nges et al., 2022, p. 4)  “It was a good… good, bad situation. But towards the end, the baby is here, and she is healthy…(...)...that’s most important” (Nges et al., 2022, p. 4) *  “The fathers stated that they had to suppress their own feelings to be able to protect and support the woman. The fathers also described the hardship in putting someone else ﬁrst when you are in a state of shock and even scared of death” (Nges et al., 2022, p. 4) *  “I can’t be tired! ...I was a little tired, but not as tired as her. And at the same time, I felt that ... it is my responsibility to make sure she will calm down. Because if I show that I am tired or that I am afraid of what will happen, then she becomes even more afraid” (Nges et al., 2022, p. 4) *  “The fathers reported that being a part of the birth was mostly to support and encourage their partners, whilst asserting their love for their women and ensuring that she was not alone” (Nges et al., 2022, p. 5)  “This was done mostly through individual research because preparatory courses were rarely offered. That notwithstanding, the fathers stated that the level of information and knowledge obtained before the childbirth never seemed to be enough, since the labour and childbirth is an unpredictable process” (Nges et al., 2022, p. 5)  “In spite of the emotional diﬃculties encountered during labour and childbirth, the fathers were not discouraged. Having found their role in the womans’ world, obtaining new knowledge and a better understanding of the values of life, they still had a positive apprehension of being a part of the process” (Nges et al., 2022, p. 5) *  “The results concerning having anxiety until the baby was born; the need for information about childbirth; ﬁnding their supportive role; as well as experiencing an emotional and learning moment that was joyful” (Nges et al., 2022, p. 5) *  “When our last son was born, I went to hospital with her because there were no women to go with her. I knew some English but my she didn’t and we had a computer translation. But, when the baby came out, they turned it off. They took my baby away and gave him shots. I thought something was wrong with him and I started crying. When the nurse saw it she asked: “Are you crying because you are happy?” and I said “No, what is wrong with my baby?” and she said “Nothing, he is getting normal care.” I wanted to hold the baby ﬁrst and say a blessing like I did back home but I didn’t know how to ask or if I could. I wish I had the opportunity to do it my way. I just want them to accept and respect us just as we are, but I didn’t know how to say it...” (Wojnar, 2015, p. 363 – 364)  “Male participants wanted to talk about it during their individual interviews to explore new ways of alleviating their wives’ pain and discomfort or admitted their wives had reconstructive surgery of the perineum” (Wojnar, 2015, p. 367)  “Maternal and paternal acculturative stress scores were signiﬁcantly correlated (r = .61, p < .001)” (Khalil et al., 2022a, p. 96)  “Our results showed that higher levels of acculturative stress reported by fathers related to higher levels of PPD symptoms reported by mothers. One of the main roles of the father in the Arab American culture is to provide ﬁnancial support and the sense of security in the family” (Riggs et al., 2016, p. 89)  “It was my ﬁrst child. I was kind of worried. I was kind of happy” (Riggs et al., 2016, p. 89)  “The majority of men were not asked by health professionals about their circumstances including ﬁnancial worries, legal and housing problems” (Riggs et al., 2016, p. 90) *  “For men in this study being involved in pregnancy, childbirth, and infant care was difﬁcult” (Riggs et al., 2016, p. 90) * |
| * extract coded to more than one theme | | |
